# The Lung Alveolar Cell (LAC) miRNome and Gene Expression Profile of the SP-A-KO Mice After Infection With and Without Rescue With Human Surfactant Protein-A2 (1A^0^)

**DOI:** 10.3389/fimmu.2022.854434

**Published:** 2022-07-01

**Authors:** Nithyananda Thorenoor, Joanna Floros

**Affiliations:** ^1^ Department of Pediatrics, College of Medicine, The Pennsylvania State University, Hershey, PA, United States; ^2^ Department of Biochemistry and Molecular Biology, College of Medicine, The Pennsylvania State University, Hershey, PA, United States; ^3^ Department of Obstetrics and Gynecology, College of Medicine, The Pennsylvania State University, Hershey, PA, United States

**Keywords:** surfactant protein A2, lung alveolar cells, alveolar macrophages, *Klebsiella pneumoniae*, miRNome, gene expression, cell cycle, TP-53

## Abstract

Human surfactant protein (SP)-A1 and SP-A2 exhibit differential qualitative and quantitative effects on the alveolar macrophage (AM), including a differential impact on the AM miRNome. Moreover, SP-A rescue (treatment) of SP-A-knockout (KO) infected mice impoves survival. Here, we studied for the first time the role of exogenous SP-A protein treatment on the regulation of lung alveolar cell (LAC) miRNome, the miRNA-RNA targets, and gene expression of SP-A-KO infected mice of both sexes. Toward this, SP-A-KO mice of both sexes were infected with *Klebsiella pneumoniae*, and half of them were also treated with SP-A2 (1A^0^). After 6 h of infection/SP-A treatment, the expression levels and pathways of LAC miRNAs, genes, and target miRNA-mRNAs were studied in both groups. We found 1) significant differences in the LAC miRNome, genes, and miRNA-mRNA targets in terms of sex, infection, and infection plus SP-A2 (1A^0^) protein rescue; 2) an increase in the majority of miRNA-mRNA targets in both study groups in KO male vs. female mice and involvement of the miRNA-mRNA targets in pathways of inflammation, antiapoptosis, and cell cycle; 3) genes with significant changes to be involved in TP-53, tumor necrosis factor (TNF), and cell cycle signaling nodes; 4) when significant changes in the expression of molecules from all analyses (miRNAs, miRNA-mRNA targets, and genes) were considered, two signaling pathways, the TNF and cell cycle, referred to as “integrated pathways” were shown to be significant; 5) the cell cycle pathway to be present in all comparisons made. Because SP-A could be used therapeutically in pulmonary diseases, it is important to understand the molecules and pathways involved in response to an SP-A acute treatment. The information obtained contributes to this end and may help to gain insight especially in the case of infection.

## Introduction

Lung diseases due to bacterial infection result in a significant increase in mortality and morbidity. The Gram-negative bacterium *Klebsiella pneumoniae* (*K. pneumoniae*) was isolated from a pneumonia patient, and it is a major source of community- and hospital-acquired respiratory infection (1–3). It is found in nature (1, 2, 4, 5) and colonizes human mucosal surfaces, such as the gastrointestinal tract and oropharynx (4–6), causing infections in a number of organs including the lung, liver, urinary tract, and others, associated with increased mortality and morbidity (7). In neonates and elderly individuals, *K. pneumoniae* infection is a major health problem (8).

In pulmonary infections due to rapid *K. pneumoniae* progress, the time interval for effective treatment is minimized (9). In humans and mouse model studies, within hours of *K. pneumoniae* infection, there are significant changes in the neutrophils at lung air spaces and pulmonary edema (10–12). The lung resident key effector cells of innate immunity, the alveolar macrophages (AMs), *via* bacterial phagocytosis can effectively eliminate the bacterial infection in the lung (13). A reduction in the number of AM cells in the lungs has been associated with a decrease in the killing of *K. pneumoniae in vivo* (14). During the early and later stages of lung infection, AM cells produce inflammatory cytokines to aid in the control of the infection (15, 16).

The hydrophilic lung surfactant proteins (SPs), SP-A and SP-D, members of the family of collectins (17), provide the first line of contact for inhaled bacteria. SP-A serves important roles in both the lung innate immunity and host defense as well as in surfactant-related functions (13, 18–21). SP-A consists of a number of functional regions/domains (21), and its C-terminal carbohydrate recognition domain is important for binding to pathogens, allergens, and others (20–24). SP-A interacts with AM and *via* this interaction modulates the function and regulation of AM (13, 25–29). SP-A may also play a role in linking innate immunity and adaptive immunity (30). In the absence of SP-A, there is a significant increase in the susceptibility to pneumonia and other types of lung injury (31–35).

In humans, SP-A is encoded by two functional genes, *SFTPA1* and *SFTPA2* (21, 36, 37), that are differentially regulated (38). These genes encode SP-A1 and SP-A2, respectively. SP-A1 and SP-A2 differ virtually in all studied aspects that include qualitative (21) and quantitative (38) differences. The qualitative differences include their differential ability to regulate the AM proteome, toponome, and many others (21, 39–43). However, in the absence of SP-A, as in SP-A knockout (KO) mice, the AM proteome profile was significantly and differentially changed in response to SP-A1 or SP-A2 protein treatment (39). Furthermore, humanized transgenic mice that each expresses SP-A1 or SP-A2 exhibited, after infection, sex differences in survival and in lung function mechanics (44, 45), and the SP-A2-expressing mouse exhibited sex-dependent AM NAD(H) redox levels (46).

miRNAs are ~22-nucleotide-long non-coding RNAs with important roles in posttranscriptional regulation usually of gene silencing of target mRNAs under various conditions (47–52). SP-A1 and SP-A2 have been shown to differentially regulate the AM (53) and the Type II cell (54) miRNome in a sex-specific manner (21). The presence of SP-A2 or the presence of both SP-A1 and SP-A2 in humanized transgenic mice resulted in significant differences in AM miRNome and miRNA-mRNA target gene regulation (53, 55). However, at a later time point, an attenuation of sex differences was observed (56), implicating an interplay of post-exposure time, sex, and SP-A genotype.

Previously, we observed 1) improved survival after treatment of SP-A-KO infected mice with SP-A, and this was independent of the time of protein treatment, i.e., prior, after, or at the same time as the infection (44); and 2) a significant change in the AM miRNome and AM gene expression profile after exposure to various insults (i.e., ozone or infection) in mice that were constitutively/chronically exposed to SP-A2 (42, 53, 56). Because innate immunity and SP-A, in particular, play an important role in mitigating infection severity, we wished to investigate mechanisms in response to acute SP-A2 treatment, as this may be relevant to cases of lung infection if one were to use SP-A-regulated miRNAs or target certain genes/pathways for therapeutic purposes.

Here, we studied the impact of infection and infection plus SP-A2 (1A^0^) protein rescue on the regulation of lung alveolar cell (LAC) miRNome and gene expression in SP-A-KO mice. The majority of LACs after 6 h following infection are AMs (~70%–75%), as assessed by differential cell count after Papanicolaou staining (see *Materials and Methods*). The KO mice of both sexes were infected with *K. pneumoniae* with or without SP-A2 (1A^0^) protein rescue. Six hours later, the expression levels of LAC miRNAs, genes, and miRNA-mRNA targets of significantly changed miRNAs as well as various pathways were studied. A number of molecules (miRNAs, miRNA-mRNA targets, and genes) were identified with significantly changed levels as a function of exposure and sex, and specific pathways were identified as being significant in these processes. The cell cycle was ubiquitous, as this was significant in all comparisons made, although the specific molecules involved differed under the different studied conditions. The pro-inflammatory pathway was another important pathway. In the gene expression study, an unexpected pathway known to play a role in cancer, the TP-53, was identified. To our knowledge, this is the first study to investigate SP-A treatment in response to infection, and the information obtained may provide useful insight, as SP-A is moving toward therapeutic considerations (57, 58).

## Materials and Methods

### Animals

Male and female SP-A-KO mice (~12 weeks old) were used in the current study. All of the animals were kept in a pathogen-free environment, as described previously (44, 45). The estrous cycle in female mice was synchronized, as previously noted (44, 45). Twenty-eight mice were used (16 for miRNA and gene expression profiling and 12 for miRNA target gene validation). The Pennsylvania State University Medical Center Institute Animal Care and Use Committee (IACUC) approved the procedures and the animal protocol (#44968) used.

### *Klebsiella pneumoniae* Preparation and Infection of Mice

*K. pneumoniae* bacteria (ATCC 43816) were obtained (Rockville, MD, USA) and prepared as described previously (32, 44, 45, 59). Approximately, 450 colony forming units (CFUs) in a 50 µl suspension were used to infect each mouse. The CFU/ml values were calculated based on the standard curve obtained by measuring the growth of bacteria at OD_660_. The mice were infected oropharyngeally (42, 60) after being anesthetized with a mixture of ketamine and xylazine as described previously (32, 44, 45, 59, 60). Male and female SP-A-KO mice (n = 4/group for miRNA and gene expression profile and n = 3/group for qRT-PCR validation) were used. Based on previous study findings (39, 42, 61, 62), the 6-h time point was selected to study LAC miRNAs and gene expression profile from KO mice in response to bacterial infection with and without rescue with SP-A. We hypothesized that the 6-h time interval would allow for the study of relatively early events.

### Treatment of Mice With SP-A2 (1A^0^) Protein

SP-A-KO mice were anesthetized, and one group of mice was infected with *K. pneumoniae* (~450 CFU/mouse) as described above. Another group was infected as described above and, at the same time, these mice received 10 µg (50 µl) of purified SP-A2 (1A^0^) protein (42, 44, 60). In this study, 10 µg of purified protein was chosen, as this was shown in a previous rescue study (44) to significantly improve the survival of infected mice. The protein used for the rescue was obtained from stably transfected CHO cell lines as described (63). The mice were monitored for 6 h after infection.

### Isolation of Lung Alveolar Cells From Infected Mice

LAC were obtained from SP-A-KO mice by bronchoalveolar lavage (BAL) at 6 h, after infection alone, and after infection plus SP-A2 (1A^0^) protein treatment as described previously (42, 61). The cells in the BAL were separated by centrifugation (150 × *g* for 5 min) (31, 53), and a total cell count was performed, and cells were frozen (-80°C) until further use for either miRNA or gene expression studies. Briefly, the cell pellet was washed with 1× PBS (Gibco, Waltham, MA, USA), and the LACs, as a whole, were used in the present study without any cell sorting. Randomly, a fraction of the cells from 2 samples from each group was used to prepare cytospins, cells were subjected to Papanikolaou staining, and a differential cell count was performed. The majority of LACs at 6 h after infection are AMs (~70%–75%). Other cells early in the response to infection may include neutrophils (10–12). SP-A treatment is not expected to change the AM cell population (39, 61).

### RNA Extraction, Library Construction, and Sequencing

Total RNA extracted from LACs was used for library construction and sequencing, as described previously (42, 55).

#### miRNA Analysis

Small RNA sequencing (RNA-seq) libraries were generated by NEXTflex Small RNA Library Prep Kit v3 for Illumina (BioO Scientific, Austin, TX, USA), followed by deep sequencing on an Illumina HiSeq 2500 as per the manufacturer’s instructions. Briefly, 1–2 ng of total RNA was ligated with chemically modified 3′ and 5′ adapters that can specifically bind to mature microRNAs, followed by reverse transcription and PCR amplification. Unique index sequence tags were introduced during PCR to enable multiplexed sequencing. Each library was assessed for the presence of desired microRNA population and approximate library quantity by Bioanalyzer High Sensitivity DNA Kit (Agilent Technologies). Pooled libraries were denatured and loaded onto a TruSeq Rapid flow cell on an Illumina HiSeq 2500 and run for 50 cycles using a single-read recipe according to the manufacturer’s instructions. De-multiplexed sequencing reads passed the default purify filtering of Illumina CASAVA pipeline (released version 1.8) and were quality trimmed/filtered using The FASTX-Toolkit (http://hannonlab.cshl.edu/fastx_toolkit). The filtered reads were further trimmed with both 5′ and 3′ adapter sequences and subjected to Chimira suite to align and count miRNA expression (64).

The differentially expressed miRNAs between male and female mice under the studied condition were identified by using edgeR test method (65) and TCC v1.14.0 R package (66) with a false discovery rate (FDR)-adjusted p-value of 0.1 as a significance cutoff for miRNA identification. Outliers and other inconsistencies were removed based on 1) the lack of good correlation of data count among the groups in at least 3 replicates and on average tag count data and 2) whether the value was higher than twice the standard deviation. The differentially expressed miRNAs (n = 178) used for further analysis were selected based on their fold change and their p-value (p < 0.05).

#### Gene Expression Analysis

QuantSeq 3′ mRNA-Seq Library Prep Kit FWD from Illumina (Lexogen, Vienna, Austria) was used to generate mRNA-Seq libraries as per manufacturer’s recommendation, followed by deep sequencing on an Illumina HiSeq-2500 as per the manufacturer’s instructions. Briefly, 0.5–1 ng of total RNA was subjected to the first cDNA strand that is initiated by oligo dT priming. The synthesis of the second cDNA strand is performed by random priming in a manner that DNA polymerase is efficiently stopped when reaching the next hybridized random primer, so only the fragment closest to the 3′ end gets captured for later indexed adapter ligation and PCR amplification. The processed libraries were assessed for fragment size distribution and quantity using a BioAnalyzer High Sensitivity DNA kit (Agilent Technologies). Pooled libraries were denatured and loaded onto a TruSeq Rapid flow cell on an Illumina HiSeq 2500 (Illumina) and run for 50 cycles using a single-read recipe (TrueSeq SBS kit v3, Illumina) according to the manufacturer’s instructions. Illumina CASAVA pipeline (released version 1.8, Illumina) was used to obtain de-multiplexed sequencing reads (fastq files) that passed the default purifying filter. These were further subjected to QuantSeq data analysis pipeline on a Bluebee genomics analysis platform (Bluebee, Cambridge, MA, USA). The differentially expressed genes between male and female mice under the studied condition were identified by using the edgeR test method (65) and TCC v1.14.0 R package (66). We chose genes for further analysis based on their p-value (p < 0.05) and their expression levels (≥2-fold change) in LACs from infected and infection plus protein rescue mice.

### Lung Alveolar Cell miRNA and Gene Data Analysis

Changes in the levels of miRNAs and gene expression after infection and infection plus SP-A2 (1A^0^) were compared. Differentially expressed miRNAs and genes in SP-A-KO male and female mice were identified (data count from 3 out of 4 mice, **Supplementary Materials 1**, **2**). The fold differences for the identified miRNAs and genes between male and female mice were determined by dividing a specific individual male miRNA or gene value by the corresponding specific female miRNA or gene value and *vice versa* for the same miRNA or gene (**Supplementary Materials 1**,**2**).

### Ingenuity Pathway Analysis

Ingenuity Pathway Analysis (IPA; www.qiagen.com/ingenuity Qiagen, Redwood City) was performed as described (42, 53, 55, 67) and used values that met the cutoff of 2-fold up and downregulation in the male and female groups in the studied conditions. IPA helped to identify miRNA-mRNA targets of the significantly changed miRNAs and signaling pathways of the miRNA targets and regulatory networks of the differentially expressed genes as well as identify “integrated” signaling networks of the significantly changed miRNAs, their targets, and the differentially expressed genes under the studied conditions.

### Validation of miRNA-mRNA Target Gene Expression

The expression levels of individual miRNA targets were validated in LACs isolated from KO male and female mice after infection alone and after infection in combination with exogenous SP-A2 (1A^0^) protein by qRT-PCR as described previously (42, 55). The expression levels of BCL2, CASP9, CCND1, CCND2, CDK7, CDKN2A, E2F1, E2F2, E2F3, EGR2, FOXO1, FOXO3, IL-6, MYC, PPARA, PPARG, SMAD2, STAT-3, TLR2, TNF, and TNFSF12 were assessed by real-time PCR using RT2 SYBR Breen ROX qPCR master mix (#330520, Qiagen) on a QuantStudio 12K Flex Real-Time PCR system (Applied Biosystems, Waltham, MA, USA) at the Pennsylvania State University College of Medicine Genomic Core Facility. The RT2 qPCR Primer assays were purchased from Qiagen. The LAC samples [3 animals/sex/treatment—infection alone or in combination with SP-A2 (1A^0^) protein] were analyzed in triplicate/animal. The glyceraldehyde 3-phosphate dehydrogenase (GAPDH) level was used, as a standard, to quantify the relative expression levels of the studied genes. The relative expression levels of genes were determined by the 2^-ΔCT^ in which ΔCT was calculated as follows: ΔCT = CT _gene-of-interest_ - CT _housekeeping gene_.

### Statistical Analysis

Significant differences of the miRNAs and gene expression levels in male and female mice after infection and after infection plus SP-A2 (1A^0^) protein were assessed by a two-tailed t-test and nonparametric Mann–Whitney test. Multiple comparison analysis was performed by one-way analysis of variance (ANOVA) followed by Bonferroni correction for multiple comparisons. A p < 0.05 was considered statistically significant. All of the data points are means ± standard deviation, and analyses were performed using GraphPad Prism software version 5.0 (GraphPad Software, San Diego, CA, USA).

## Results

### Lung Alveolar Cell miRNome

#### Effect of Infection and Infection Plus Exogenous SP-A2 (1A^0^) Protein Rescue on Lung Alveolar Cell miRNome

**Figure 1:**
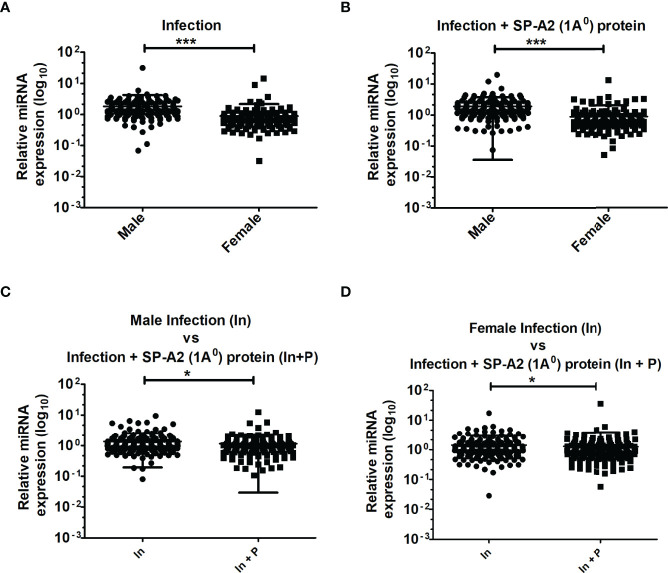
LAC miRNome in KO males and females mice after *K*. *pneumoniae* infection and infection plus SP-A2 (1A^0^) protein rescue. Comparisons between miRNAs identified in KO, M vs. F, after infection **(A)**, M vs. F, after infection plus SP-A2 (1A^0^) protein rescue **(B)**. Male mice: infection vs. infection plus SP-A2 (1A^0^) protein **(C)**. Female mice: infection vs. infection plus SP-A2 (1A^0^) protein **(D)**. Significant differences were observed between sexes and between bacterial infection and infection plus rescue (**A–D**; p < 0.05). *p < 0.05, ***p < 0.001. In; Infection, In + P; infection plus SP-A2 (1A0) protein.

A total of 178 LAC miRNAs were found in male and female mice of both study groups (**Supplementary Material 1**). A two-tailed t-test and nonparametric Mann–Whitney test were used to assess differences (p < 0.05) (**Figures 1A–D**). No significant differences were observed in any of the comparisons by one-way ANOVA or Bonferroni multiple comparison analysis (data not shown).

#### miRNAs That Changed ≥2-Fold After Infection and Infection Plus SP-A2 (1A^0^) Protein Rescue

We next studied LAC miRNAs that exhibited ≥2-fold changes in response to infection vs infection plus SP-A2 (1A^0^) protein rescue in KO male and female mice.. A comparison of LAC miRNAs from infected KO male and female mice revealed 1) 47 miRNAs in male mice and 7 miRNAs in female mice in response to infection and 2) 53 miRNAs in male mice and 12 miRNAs in female mice after infection plus SP-A2 (1A^0^) (**Supplementary File 1**). Another comparison of LAC miRNAs that changed in response to infection vs. infection plus SP-A2 (1A^0^) protein rescue in the same sex revealed that, in male mice, 25 miRNAs were differentially expressed (≥2-fold) in response to infection compared to 12 miRNAs in response to infection plus SP-A2 (1A^0^) protein, and in female mice, 26 miRNAs were differentially expressed (≥2-fold) in response to infection compared to 15 miRNAs in response to infection plus SP-A2 (1A^0^) protein rescue (**Supplementary File 1**). In both comparisons, miRNAs with ≥2-fold expression level were specific to infection or infection plus SP-A2 (1A^0^) protein rescue. No miRNA was found to be in common in either sex between the two conditions (**Supplementary File 1**).

#### Ingenuity Pathway Analysis

**Figure 2:**
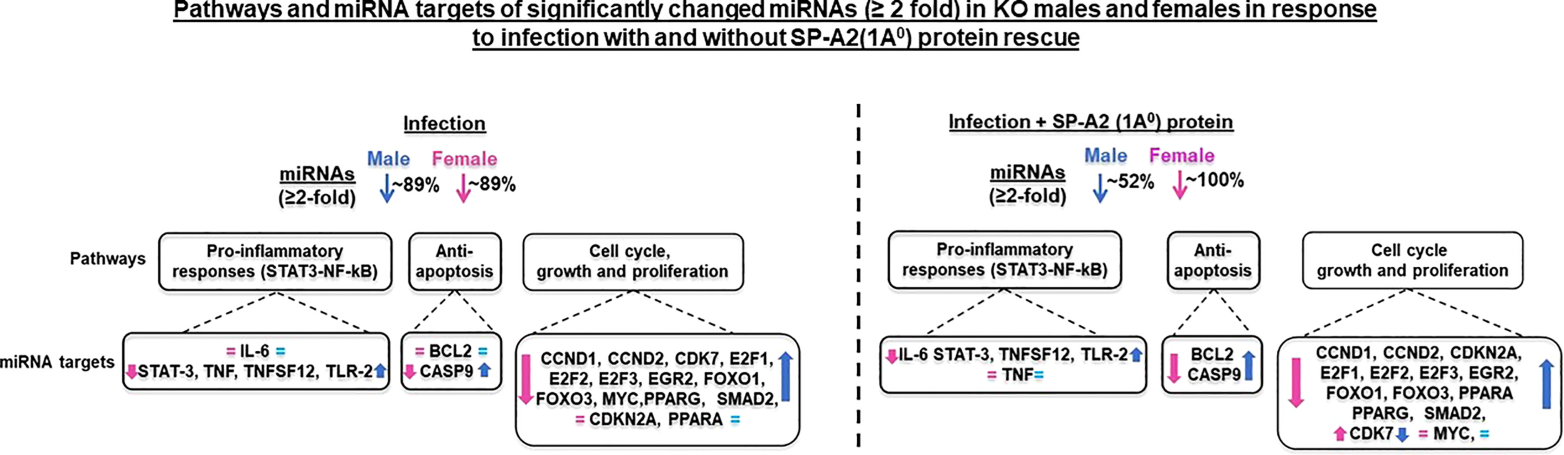
Effect of infection and infection plus SP-A2 (1A^0^) protein rescue on the miRNome, miRNA-mRNA targets, and pathways of the LAC KO male and female mice. A comparison is shown of the miRNAs, target genes, and pathways of the LAC KO after infection and infection plus SP-A2 (1A^0^) rescue in the left and right panels, respectively. The significantly regulated miRNAs (n = 19) each present in KO male and female groups in response to infection were largely decreased by ~89% in male (blue arrow) and female (pink arrow) mice. In the infection plus SP-A2 (1A^0^) protein rescue, ~52% of the significantly regulated miRNAs (n = 19) were decreased in male (blue arrow) and 100% in female (pink arrow) mice; a few miRNAs showed an increase **(Table 1)**). Three pathways are depicted as assessed by IPA of the significantly changed miRNAs. The upregulated miRNA-mRNA targets in KO male mice (blue arrow) and the downregulated targets in female mice (pink arrows) are depicted after infection and infection plus rescue in the left and right panels, respectively. The mRNA targets that did not change after infection and infection plus SP-A2 (1A^0^) protein are shown in pink and blue equal sign.

IPA was used to assess biological functions and miRNA targets of significantly changed miRNAs. The miRNA-mRNA targets of the significantly changed miRNAs and the signaling pathways that these are involved in are shown in **Figure 2**. The miRNA-mRNA targets include BCL2, CASP9, CCND1, CCND2, CDK7, CDKN2A, E2F1, E2F2, E2F3, EGR2, FOXO1, FOXO3, IL-6, MYC, PPARA, PPARG, SMAD2, STAT-3, TLR2, TNF, and TNFSF12. The miRNAs that were changed significantly in KO male and female mice under the studied conditions and their targets are listed in **Table 1**.

**Table 1:** LAC KO miRNA levels in male and female mice and their mRNA targets after infection and infection plus SP-A2 (1A^0^) rescue.

miRNA ID	Infection		Infection + SP-A2 (1A^0^) Protein		^#^Target molecule
	Fold change				
	Male mice	Female mice	Male mice	Female mice	
let-7a-5p	1.16*	0.86*	1.64*	0.61*	CCND1, CCND2, CDKN2A, E2F1, E2F2, E2F3, MYC, PPARA, TNF, TNFSF12
miR-16-5p	0.83*	1.21*	1.39*	0.72*	BCL2, CCND1, CCND2,CDK7, E2F1, E2F2, E2F3, TNFSF12
miR-17-5p	2.09	0.48*	1.67*	0.60*	CCND1, CDK7, E2F1, E2F2, E2F3, EGR2, MYC, STAT-3, PPARA, TNFSF12,
miR-22-3p	0.44*	2.28	1.32*	0.76*	E2F1, E2F3
miR-23a-3p	1.23*	0.81*	1.98	0.51*	E2F1, E2F3,TNFSF12
miR-30c-5p	1.74*	0.57*	1.64*	0.61*	PPARA
miR-34a-5p	1.30*	0.77*	3.49	0.29*	PPARA, PPARG
miR-103-3p	0.80*	1.24*	1.38*	0.72*	E2F1, E2F3, PPARA
miR-125b-5p	1.10*	0.91*	1.48*	0.68*	TLR2
miR-143-3p	0.51*	1.94	0.73*	1.38*	E2F1, E2F3, PPARA
miR-155-5p	2.45	0.41*	3.01	0.33*	IL-6, TLR2, TNF
miR-181a-5p	1.09*	0.91*	2.44	0.41*	SMAD2
miR-182-5p	1.08*	0.93*	1.53*	0.66*	FOXO1, PPARA
miR-185-5p	0.70*	1.42*	2.18	0.46*	SMAD2
miR-191-5p	1.20*	0.83*	2.10	0.48*	IL-6
miR-200b-3p	1.15*	0.87*	1.28*	0.78*	PPARA
miR-221-3p	0.96*	1.04*	2.17	0.46*	E2F1, E2F2, E2F3
miR-378a-3p	1.27*	0.79*	2.37	0.42*	CASP9, FOXO3, PPARA
miR-423-5p	1.94	0.52*	3.11	0.32*	SMAD2

^*^Indicates downregulation. ^**#**^Identified by IPA.

#### miRNA-mRNA Target Validation by qRT-PCR Analysis

**Figure 3:**
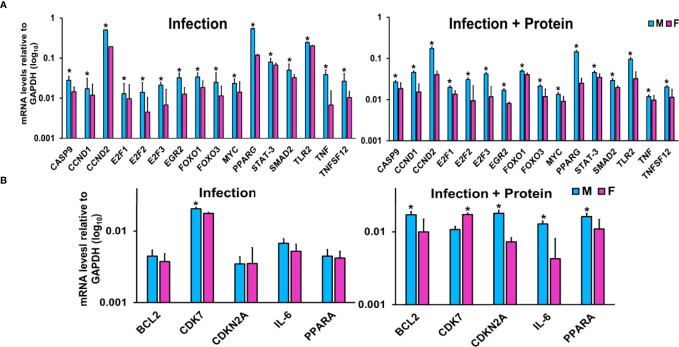
Effect of infection and infection plus SP-A2 (1A^0^) protein rescue on miRNA-mRNA targets. Panels **A, B** show the gene expression levels in KO male and female mice. Significant sex differences after infection in the expression levels of CASP9, CCND1, CCND2, E2F1, E2F2, E2F3, EGR2, FOXO1, FOXO3, MYC, PPARG, SMAD2, STAT-3, TLR2, TNF, and TNFSF12 (upregulated in male mice compared to female mice) are shown in panel **(A)** on the left. The expression levels of BCL2, CDK7, CDKN2A, IL-6, and PPARA after infection that exhibited no changes between sexes in response to infection except CDK7 (showed a significant increase in male mice) are shown in panel **(B)** on the left. However, in response to infection plus protein rescue, all of the genes exhibited an increase in male vs. female mice (**A, B** on the right) except the CDK7 that exhibited an increase (p < 0.05) in female mice (**B** on the right). The expression levels of specific mRNA targets were normalized to GAPDH and are depicted by blue and pink bars, respectively, for male and female mice. An asterisk (*) marks the differences (p < 0.05) between male and female mice.

LACs derived from mice of either sex from the two studied conditions were used for this analysis (**Figure 3**). The expression levels of CASP9, CCND1, CCND2, E2F1, E2F2, E2F3, EGR2, FOXO1, FOXO3, MYC, PPARG, SMAD2, STAT-3, TLR2, TNF, and TNFSF12 were significantly upregulated in KO male vs. female mice under both studied conditions (**Figure 3A**). On the other hand, the expression levels of BCL2, CDKN2A, IL-6, and PPARA remained similar in both male and female mice after infection but were significantly upregulated in male mice after infection in combination with exogenous SP-A2 (1A^0^) protein (**Figure 3B**). The expression level of CDK7 was significantly increased in male mice after infection but decreased significantly compared to female mice in response to infection in combination with exogenous SP-A2 (1A^0^) protein (**Figure 3B**).

### Gene expression of Lung Alveolar Cells From Infected Mice and Infected Plus SP-A2 (1A^0^) Protein Rescue Mice

#### Ingenuity Pathway Analysis

**Figure 4:**
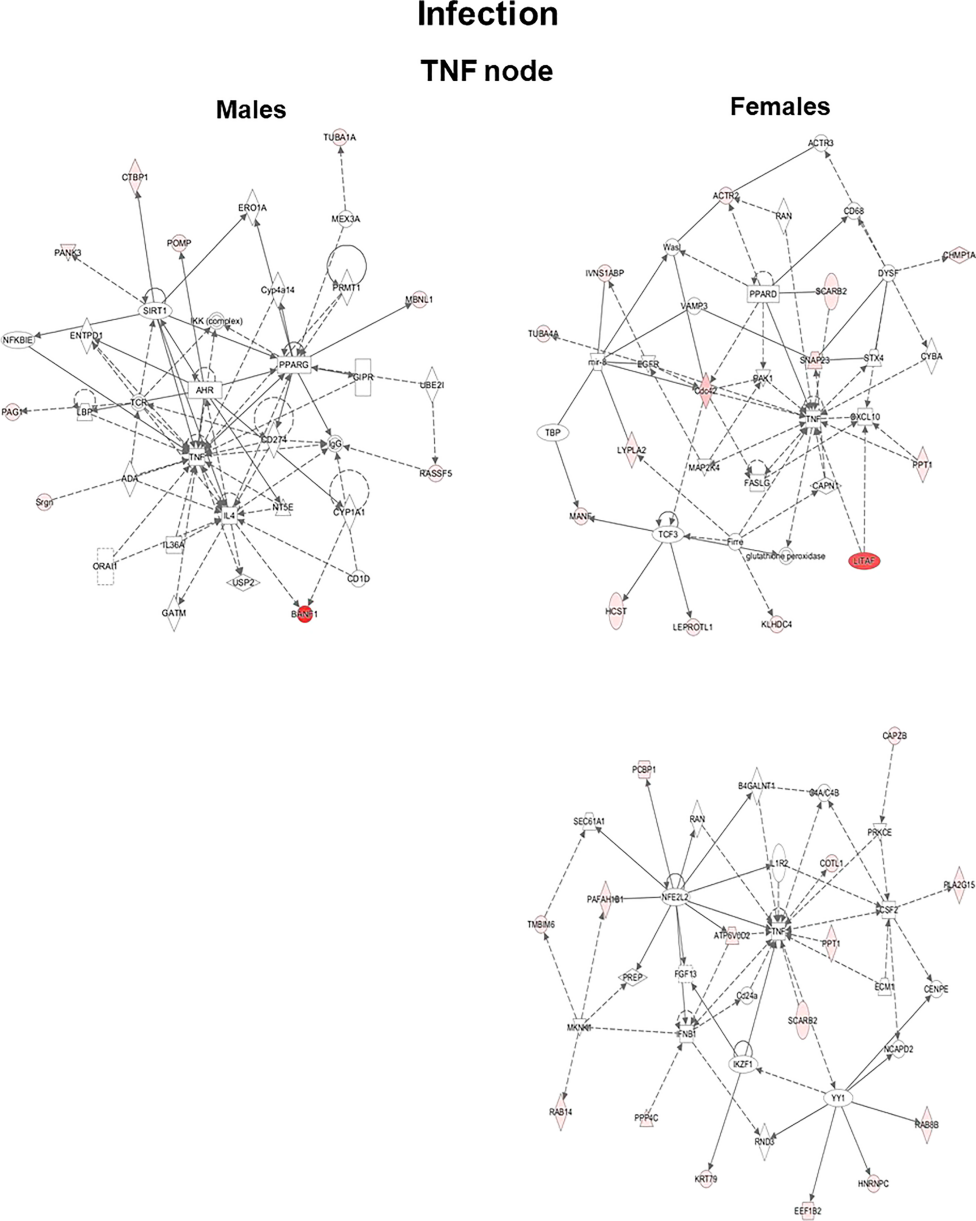
Ingenuity pathway analysis. Biological networks for the TNF signaling of genes with ≥2-fold changes in their expression level are shown for the KO LAC of both sexes at 6 h after infection. Direct and indirect gene interactions are marked with solid and dashed lines, respectively. Networks on the left and right show one pathway for male mice and two pathways for female mice, respectively. The different shapes depict different functional classes as follows: Square and concentric (double) circles denote cytokines and complex/groups, respectively. Diamonds denote peptidases and enzymes. Ovals denote transmembrane receptors and transcription regulators; triangle, kinases and phosphatases; rectangles, ion channels, G-protein-coupled receptors, and ligand-dependent nuclear receptors; and trapezoids, microRNAs and transporters.

The genes that exhibited significant changes (≥2-fold) in response to infection and infection plus SP-A2 (1A^0^) protein between KO male and female mice were used for IPA. Three signaling nodes, tumor necrosis factor (TNF), TP-53, and cell cycle, were identified where each node had direct interactions with 4 or more molecules in the studied conditions. The TNF node even though lacked direct interactions with 4 or more molecules is shown in **Figure 4** because a large number of genes (n = 8) with ≥2-fold change had indirect interactions. The functional relationship plots of the signaling nodes in both sexes in KO under the studied conditions are presented in **Figure 4** and **Supplementary Figure 1**. These together show that many of the observed genes with ≥2-fold changes were previously shown to contribute, *via* either direct (solid lines) or indirect (dashed lines) interactions, to TNF, cell cycle, and TP-53 signaling nodes under the studied conditions (**Figure 4**, **Supplementary Figure 1**).

#### Differentially Expressed Genes in Knockout Lung Alveolar Cells From Mice Under the Studied Conditions, Infection and Infection Plus Rescue

We identified differentially expressed genes (p < 0.05) for several of the comparisons made. These include a) M vs. F, infection, n = 169 genes; b) M vs. F, infection plus SP-A2 (1A^0^) protein, n = 105 genes; c) male mice, infection vs. infection plus SP-A2 (1A^0^), n = 245 genes; and d) female mice, infection vs. infection plus SP-A2 (1A^0^), n = 188 genes (**Supplementary File 2**).

Next, we assessed changes in specific genes that either increased (≥2-fold) or decreased (≤2-fold) significantly in either sex under each studied condition. After infection, out of the 169 significantly changed genes (≥2-fold), 38 were increased and 131 were decreased in male vs. female mice and *vice versa* (**Supplementary File 2**). In response to infection plus SP-A2 (1A^0^), out of the 105 significantly changed genes (≥2-fold), 22 and 84 had increased and decreased levels, respectively, in male vs. female mice and *vice versa* (**Supplementary File 2**). In male mice, out of 245 significantly changed genes (≥2-fold), 178 and 65 had increased and decreased levels, respectively, in response to infection vs. infection plus SP-A2 (1A^0^) and *vice versa* (**Supplementary File 2**). Whereas in female mice, out of the 188 significantly changed genes, 127 and 61 exhibited increased and decreased levels, respectively, in response to infection vs. infection plus SP-A2 (1A^0^) and *vice versa* (**Supplementary File 2**). Next, we compared genes identified under the studied condition in male and female mice and found 45 genes to be in common under all studied conditions (**Table 2**).

**Table 2:** Relative content (≥2-fold or ≤2-fold) of genes found to be in common in KO male vs. female (M/F) mice and *vice versa* after infection and infection plus SP-A2(1A^0^) rescue are shown.

Gene Symbol	Fold change (≥2 or ≤2)					
	Male mice			Female mice		
	Infection	Infection plus Protein	p-value	Infection	Infection plus Protein	p-value
Anxa2	10.500	0.095	0.012	14.943	0.067	0.005
App	9.888	0.101	0.019	23.387	0.043	0.002
Arl6ip1	48.327	0.021	0.001	7.220	0.138	0.040
Atp6v1b2	11.270	0.089	0.040	7.451	0.134	0.033
B2m	7.368	0.136	0.046	14.988	0.067	0.007
Cd47	15.293	0.065	0.027	0.003	391.712	0.000
Cd63	36.338	0.028	0.004	11.236	0.089	0.009
Cd74	42.953	0.023	0.001	9.876	0.101	0.012
Cebpb	8.023	0.125	0.025	15.958	0.063	0.007
Chmp1a	0.112	8.899	0.012	11.532	0.087	0.024
Chmp4b	0.143	6.990	0.009	44.313	0.023	0.000
Cotl1	8.627	0.116	0.031	7.323	0.137	0.028
Cox6a1	13.444	0.074	0.023	43.058	0.023	0.004
Ctsz	101.999	0.010	0.000	10.138	0.099	0.020
Dazap2	15.871	0.063	0.013	64.249	0.016	0.000
Ear2	14.074	0.071	0.007	6.663	0.150	0.026
Furin	18.289	0.055	0.012	41.871	0.024	0.001
Gm10076	15.460	0.065	0.015	11.796	0.085	0.019
Gm23935	146.087	0.007	0.000	52.072	0.019	0.001
Gpx1	24.106	0.041	0.004	29.036	0.034	0.000
Grn	17.153	0.058	0.006	24.312	0.041	0.001
Iqgap1	7.248	0.138	0.048	7.978	0.125	0.029
Itgb1	18.834	0.053	0.024	28.461	0.035	0.010
Itm2c	19.639	0.051	0.016	156.657	0.006	0.000
Lcp1	12.632	0.079	0.013	33.804	0.030	0.001
Lsp1	0.113	8.849	0.042	8.749	0.114	0.045
Pafah1b1	0.084	11.933	0.011	40.320	0.025	0.007
Pla2g15	36.258	0.028	0.007	6.479	0.154	0.045
Psap	45.531	0.022	0.000	38.107	0.026	0.001
Ptprc	13.997	0.071	0.040	17.303	0.058	0.006
Rab14	11.907	0.084	0.040	12.096	0.083	0.004
Rpl36a	42.542	0.024	0.002	16.947	0.059	0.005
Rpl37	8.835	0.113	0.041	11.783	0.085	0.017
Rps25	38.956	0.026	0.000	12.910	0.077	0.009
Rps3	18.131	0.055	0.014	54.118	0.018	0.001
Rps5	7.865	0.127	0.042	9.942	0.101	0.017
Rps9	7.408	0.135	0.045	30.685	0.033	0.001
Shisa5	33.361	0.030	0.002	49.892	0.020	0.000
Tmbim6	7.950	0.126	0.031	24.143	0.041	0.001
Tmed10	84.217	0.012	0.001	13.154	0.076	0.035
Tubb5	14.562	0.069	0.013	49.324	0.020	0.001
Txnip	10.103	0.099	0.033	51.432	0.019	0.000
Ube2d3	17.291	0.058	0.024	29.690	0.034	0.006
Vdac2	0.116	8.650	0.016	0.121	8.268	0.040
Wfdc21	148.952	0.007	0.000	10.742	0.093	0.026

#### Sex and Treatment Differences in Lung Alveolar Cell Gene Expression After Infection and After Infection Plus SP-A2 (1A^0^) Protein

**Figure 5:**
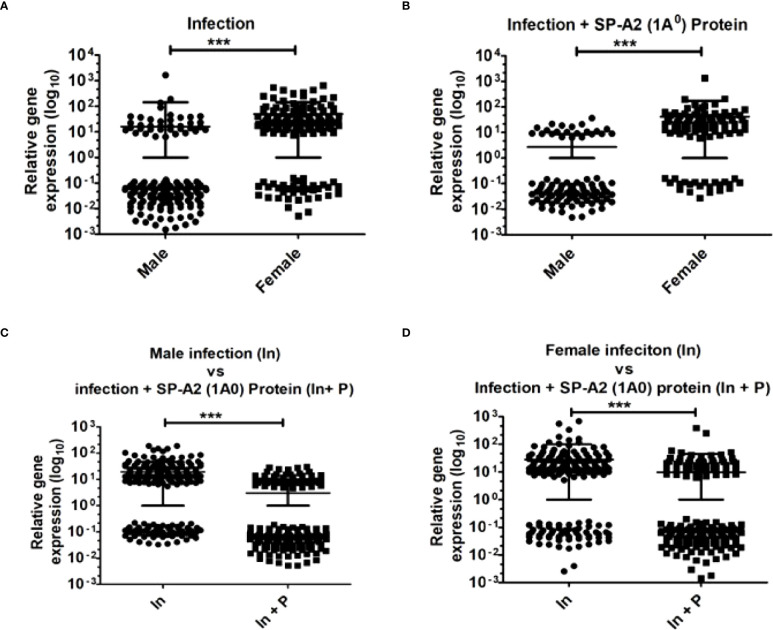
Gene expression in KO mice of both sexes at 6 h after *K. pneumoniae* infection and infection plus SP-A2 (1A^0^) protein rescue. A total of 169 genes in M vs. F after infection **(A)**, 105 genes in M vs. F after infection plus SP-A2 (1A^0^) protein rescue **(B)**, 245 genes in male mice of infection vs. infection plus SP-A2 (1A^0^) protein rescue groups **(C)**, and 188 genes in female mice of infection vs. infection plus SP-A2 (1A^0^) protein rescue groups **(D)** were changed significantly and used in these analyses. These comparisons (n = 4/group) were significant (p < 0.05). Sex and treatment differences (***p < 0.001) were observed under studied conditions.

The two-tailed t-test and nonparametric Mann–Whitney U-test were used to study the relative expression levels (p < 0.05) of genes in the LAC under the studied conditions. The results showed significant differences both as a function of sex and treatment after infection or infection plus SP-A2 (1A^0^) protein (**Figures 5A–D**).

### Significant Pathways After Integration of miRNAs, miRNA-mRNA Target Genes, and Genes Expressed Under the Studied Conditions

**Figure 6:**
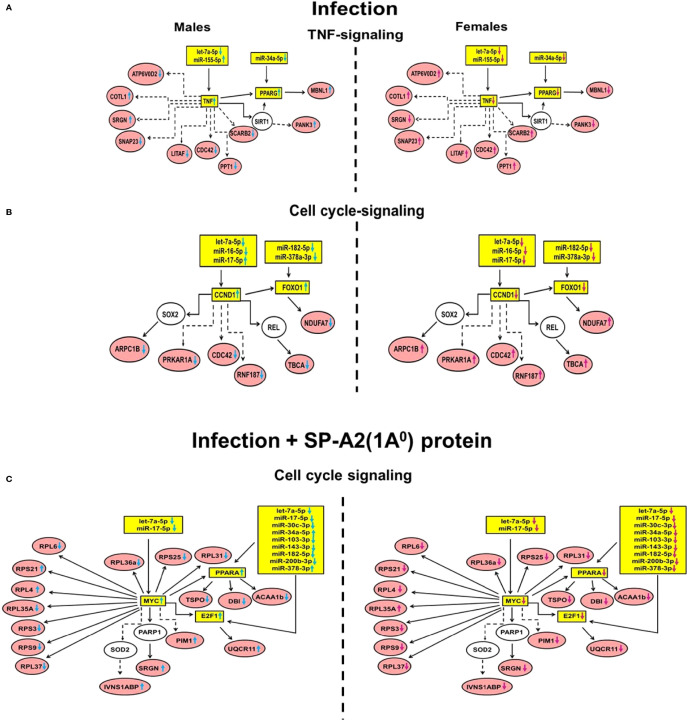
Significant pathways that involve miRNAs, miRNA-mRNA targets and genes expressed following infection (Panel **A, B**) and infection plus SP-A2 (1A0) protein rescue (Panel **C**) are shown for males (left side diagrams) and for females (right side diagrams). Interactions of miRNA targets with genes identified from the gene expression analysis are shown. The molecules in these interactions are involved in the TNF and cell signaling pathways (p < 0.05). The miRNAs and their targets validated by qRT-PCR are highlighted in yellow. The genes that interact with miRNA targets are highlighted in red, and genes not identified in our study but exhibited interactions with the identified genes are shown in white. Direct and indirect interactions are shown with solid and dashed lines, respectively. The up and downregulation of miRNAs and genes are shown in blue and pink colored arrows indicating males and females, respectively.

In response to infection and infection plus SP-A2 (1A^0^) protein rescue, a subset of the miRNAs, miRNA-mRNA targets, and genes identified with significant changes in their levels in male and female mice were involved in TNF and cell cycle signaling pathways (**Figure 6**). These are referred to as “integrated” pathways in the *Discussion*.

#### In Response to Infection

Two signaling pathways were identified, the TNF and the cell cycle. 1) In TNF signaling, the expression level of miRNAs let-7a-5p, miR-34a-5p, and miR-155-5p was downregulated (p < 0.05) in both sexes, except miR-155-5p that showed a significant increase in male mice. The identified miRNAs were predicted to bind and regulate TNF and PPARG, and their altered expression was associated with an increase in the expression (p < 0.05) of these target molecules in male vs. female mice (**Figure 6A**). IPA revealed that several of the identified genes in the gene expression study (ATP6V0D2, COTL1, SRGN, SNAP23, LITAF, CDC42, PPT1, SCARB2, and PANK3) have an indirect interaction with the miRNA targets especially TNF, whereas the MBNL1 has a direct interaction with PPARG (**Figure 6A**). The expression of the ATP6V0D2, SNAP23, LITAF, CDC42, PPT1, and SCARB2 genes was downregulated (p < 0.05), and the expression of SRGN, PANK3, and MBNL1 was upregulated (p < 0.05) in male vs. female mice. However, the expression of the COTL1 gene was upregulated (p < 0.05) in both sexes (**Figure 6A**).

2) In cell cycle signaling, the expression level of miRNAs let-7a-5p, miR-16-5p, miR-17-5p, miR-182-5p, and miR-378a-3p was downregulated (p < 0.05) in male and female mice, except miR-17-5p that showed a significant increase in male mice. These miRNAs were predicted to bind and regulate CCND1 and FOXO1. These target molecules showed a significant increase in their expression in male vs. female mice (**Figure 6B**). IPA revealed that several of the identified genes (ARPC1B, PRKAR1A, CDC42, RNF187, and TBCA) have indirect interaction with the miRNA target CCND1, and the NDUFA7 gene has a direct interaction with FOXO1 (**Figure 6B**). The significantly changed genes were downregulated (p < 0.05) in male vs. female mice (**Figure 6B**).

#### In Response to Infection Plus SP-A2 (1A^0^) Protein Rescue

One signaling pathway was identified under this condition, namely, the cell cycle. In this pathway, the expression level of miRNAs let-7a-5p, miR-17-5p, miR-30c-3p, miR-34a-5p, miR-103-3p, miR-143-3p, miR-182-5p, miR-200b-3p, and miR-378a-3p was significantly downregulated in male and female mice, except miR-34a-5p and miR-378-3p. These two showed a significant increase in male mice. These miRNAs were predicted to bind and regulate MYC, E2F1, and PPARA; these target molecules showed an increase (p < 0.05) in their expression in male vs. female mice (**Figure 6C**). IPA revealed that several of the identified genes (RPL6, RPS21, RPL4, RPL35A, RPS3, RPS9, RPL37, RPL36a, RPS25, RPL31, TSPO, DBI, ACAA1b, and UQCR11) have direct interaction with the miRNA-mRNA targets, i.e., MYC, E2F1, and PPARA, whereas PIM1, SRGN, and IVNS1ABP have indirect interaction with MYC (**Figure 6C**). The expression level of RPS21, RPL4, UQCR11, SRGN, and IVNS1ABP genes was upregulated (p < 0.05) in male vs. female mice, but the expression of RPL35A was upregulated (p < 0.05) in female vs. male mice (**Figure 6C**). The expression of the other genes that interacted with MYC, E2F1, and PPARA was downregulated in both sexes under the studied condition. Together, these may provide some insight into the sex-specific response in the presence or absence of SP-A, observed here and in other studies, following bacterial infection.

## Discussion

AMs are key effector cells in the innate immunity of the lung, and their regulation and function can be affected by SP-A (13, 20, 21, 32, 59, 68–70). Several studies have shown in response to *K. pneumoniae* infection differences in survival as a function of sex in various types of mice including wild-type, SP-A-KO, and humanized transgenic mice, where each mouse line expresses a different human SP-A1 and SP-A2 variant (21, 32, 44, 59). In fact, the rescue of SP-A-KO mice with exogenous SP-A has been shown to significantly improve survival after bacterial infection regardless of whether the SP-A treatment occurred before or after infection or simultaneously with infection (44). Human SP-A1 and SP-A2 variants exhibit sex differences in their ability to regulate *in vivo* the miRNome (21) of AM (53) and Type II cells (54). Here, we studied the effect of *K. pneumoniae* infection and infection plus SP-A2 (1A^0^) protein rescue on the differential regulation of the LAC miRNome and gene expression of SP-A-KO mice. Mice were infected with *K. pneumoniae* or infected plus rescue with SP-A2 (1A^0^) protein. LAC miRNAs and gene expression levels were studied at 6 h after infection and infection plus rescue. The miRNA-mRNA target genes and signaling networks of the significant miRNAs were studied by IPA and validated by qRT-PCR and the gene expression profile by IPA. We observed the following: 1) differences (p < 0.05) in the LAC miRNome and gene expression of KO as a function of sex and condition; 2) significant increases in the overwhelming majority of miRNA target genes in KO male mice in response to infection and infection plus rescue; 3) involvement of the miRNA-mRNA targets in various pathways that included pathways involved in inflammation, antiapoptosis, and cell cycle; 4) based on LAC gene expression, signaling pathways of TP-53, TNF, and cell cycle signaling nodes were identified; and 5) miRNA-mRNA target and gene expression was significantly increased in KO male mice compared to female mice. A subset of the significantly changed targets, genes, and miRNAs was connected *via* the TNF and cell cycle signaling pathways in response to infection and the cell cycle signaling pathway alone in response to infection plus SP-A2 (1A^0^) protein rescue.

The role of sex and sex hormones on lung immunity in both humans and animals has been previously documented (71–81). A number of animal models have shown differences in survival after infection as a function of sex (13, 21, 32, 44, 59, 82) as well as in disease susceptibility and severity (32, 44, 59, 83–87). In humans, both prematurely born males vs. females exhibit higher susceptibility to neonatal respiratory distress syndrome (RDS) (73, 74), and adult males exhibit a higher susceptibility in Idiopathic pulmonary fibrosis (IPF), and Chronic obstructive pulmonary disease (COPD) (77, 88) and others, as well as in different types of pneumonia (79, 80, 88, 89).

In the current study, we found significant differences in the LAC miRNome, gene expression, and miRNA-mRNA target gene expression in KO, in terms of sex, under the studied conditions. An interesting and consistent observation was made, as also observed in previous related studies (42, 55, 56). The upregulation and downregulation of the majority of miRNAs, miRNA-mRNA targets, and genes were largely opposite in male and female mice, and the relevant discussion below pertains primarily to male mice. The sex differences observed are not surprising, as these have been observed before with other mouse models (13, 21). However, the present study, apart from contributing to a strong foundation that sex is an important variable that cannot be ignored, is the first study where the miRNome of infected mice was studied using mice that were never exposed to SP-A until the time of experimentation. In the published miRNome studies, the mice were exposed to ozone (and not to infection as in the current study) and were chronically exposed to SP-A. The available data indicate that the sex variable is important regardless of the exposure insult (i.e., ozone exposure, infection, others) and the studied conditions (13, 21). Although the detailed mechanisms for this sex-dependent regulation are not entirely clear, the collective literature points to a need of taking sex into consideration in study design and decisions that may impact biological processes. Moreover, the significantly changed molecules, whether miRNAs, miRNA-mRNA targets, or genes alone, were found to be involved in various signaling pathways. A subset of all three types of molecules whose expression was studied (miRNAs, miRNA-mRNA targets, and genes) converged in namely the TNF and cell cycle signaling pathways. We refer to these two pathways as “integrated’ pathways.

### Integrated Pathways

#### Cell Cycle Signaling in Response to Infection

The expression levels of both CCND1 and FOXO1 were increased in KO male mice, and the significant miRNAs shown to target these genes were for the most part downregulated compared to female mice. In the current study, the miRNAs that target CCND1, i.e., let-7a-5p, miR-16-5p, and miR-17-5p, may play a role in its regulation (90–93). CCND1 contributes to the regulation of G1-S phase transition, and the expression of CCND1 is induced by various stimuli (94, 95). The increase of CCND1 in KO male mice in response to infection may benefit LAC growth and G1-S phase progression (95), and decreased expression of CCND1 in female mice may contribute to the inhibition of cellular proliferation by a mechanism yet to be defined.

The expression level of FOXO1 as noted above was increased in KO male mice compared to female mice, and the miRNAs that target FOXO1, miR-182-5p, and miR-378-3p (**Table 1**) were significantly downregulated. FOXOs are transcription factors and may serve as a negative feedback loop in the control of cellular ROS homeostasis (96). FOXOs regulate different genes in different cell types (97) and activate the stress resistance genes and proapoptotic genes in response to different stimuli (97). Thus, the upregulation of FOXO1 may be a mechanism to alleviate stress-induced damage on LACs in KO male mice compared to female mice in response to infection. Of interest, previous studies (reviewed in Floros et al. (21)) have indicated that AMs from KO mice (i.e., AMs not exposed to SP-A) may be in a state of oxidative stress. Furthermore, a redox imaging study of a comparison of the KO AM redox status with AMs from humanized transgenic mice expressing a human SP-A transgene showed the KO AMs to be more oxidized after *in vivo* exposure of mice to ozone (46). It is possible that a similar mechanism is involved in response to infection, and FOXO1 plays a protective role in this. FOXO1, as did CCND1 (discussed above), was detected only in the infection group and not in the infection plus rescue. However, in the rescue group, one of the miRNAs (miR378a-3p) (**Table 1**) that targets FOXOs was increased, indicating that in KO LACs, additional/varied pathways may contribute to the regulation of this gene family.

#### Cell Cycle Signaling in Response to Infection Plus SP-A Protein Rescue

Even though this pathway was significant in both response to infection and response to infection plus rescue, different cell cycle molecules were significant in each. In the rescue group, three miRNA-mRNA targets (MYC, E2F1, and PPARA) were identified as targets for several miRNAs and potential regulators of several genes involved in this pathway. MYC gets activated in the G1 phase of cell growth and may serve, along with CCND1, as a G1-S phase transition regulator. The expression of E2F1 is induced by MYC (98, 99). The levels of both E2F1 and MYC were increased in KO male mice in the presence of SP-A2 (1A^0^) protein rescue at the time of infection, even though most (except miR-378-3p, in KO male mice) of the miRNAs that target these genes were decreased in both sexes.

#### TNF Signaling in Response to Infection

The “integrated” TNF pathway was identified only for the infection group and not for the infection plus rescue. This pathway in both male and female mice contained two target genes, the TNF and PPARG. SP-A is shown to modulate TNF expression in AMs (28) and in a macrophage-like cell line (100) *via* NF-κB activation/signaling (101, 102). In the latter, NF-κB inhibitors were shown to inhibit the SP-A-dependent TNF increase (101). In the absence of SP-A, TNF production was reduced (103) and an altered NF-κB pathway may play a role in SP-A-mediated TNF regulation after ozone exposure (103), a condition shown to reduce significantly the activity of SP-A (104). Whether infection in the absence of SP-A modulates TNF expression *via* different or modified NF-κB-mediated pathways, as shown previously under other conditions, is currently unknown. Although two miRNAs were identified to target TNF, the fact that their level was increased (miR-155-5p) and decreased (let-7a-5p) provides at present little insight into potential miRNA-mediated mechanisms without further experimentation.

However, upregulation of TNF may have an impact on NF-κB signaling by enhancing its nuclear translocation, which is key for NF-κB-mediated transcription of genes necessary to combat infection. In KO male mice, the IKK complex that may be activated indirectly by the TNF is increased at 6 h post-infection with or without rescue (**Figure 3**). In unstimulated cells, IKK molecules are associated with NF-κB to retain it in the cytoplasm. A variety of stimuli that include bacterial products could posttranslationally modify, *via* phosphorylation, the IKK molecules (105). This in turn initiates their destruction, which is key to freeing the NF-κB to enable its move to the nucleus in order to modulate transcription of various genes that are necessary to combat infection (105, 106). In addition, phosphorylation of IKK triggers MAPK signaling pathways, ERK1/2, JNK, and p38 (107). It would be of interest to investigate whether TNF induces prosurvival NF-κB and MAPK-dependent signaling in the studied infection models.

Furthermore, the peroxisome proliferator-activated receptors (PPARs) are transcriptional factors and members of the nuclear hormone receptor superfamily (108). One of its members, PPARG, is upregulated in the integrated pathway, and the miRNA (miR-34a-5p) that targets it is downregulated, pointing to a potential miRNA-mediated regulation of this gene in KO male mice. PPARs play a crucial role in anti-inflammatory activities in AMs (109, 110), and PPARG ligands significantly reduce cytokine production including TNF-α in human and mouse AMs (111, 112). However, the exact mechanism involved in the present model is yet to be determined.

In summary, the information from the integrated pathways that connects the three molecules (miRNAs, miRNA-mRNA targets, and genes) whose expression changed significantly shows that the general cell cycle signaling is important in both groups of study, although the actual molecules involved in each study group differ. This indicates that the presence or absence of SP-A is a key factor in the specificity of the overall process. The TNF signaling, on the other hand, was present only in the infection group. As noted above and reviewed elsewhere (21), the KO AM, which is the predominant cell in LACs, may exhibit certain deficits. Proteomics studies have shown that the proteomics profile of AM KO differs from that of wild type at baseline (113) and in response to infection (114). It is possible that the TNF signaling is necessary, as it may enable the AM KO or other cells in LACs to overcome potential inherent deficits.

### Knockout Lung Alveolar Cell miRNome After Infection or After Infection Plus Rescue With SP-A

The cell cycle signaling and a pro-inflammatory pathway were identified as being important in response to infection and infection plus rescue when the miRNA-mRNA targets were analyzed by IPA. Both of these pathways involved several of the molecules whose expression changed significantly. The cell cycle was a ubiquitous pathway, as it was significant in all comparisons made. The overwhelming majority of changed miRNAs were downregulated after infection and infection plus SP-A2 (1A^0^) protein rescue, and these were predicted to target genes that play a role in cell cycle and growth and proliferation pathways, such as CCND1, CCND2, CDK7, CDKN2A, E2F1, E2F2, E2F3, and MYC (**Table 1**). For example, miR-16-5p and miR-17-5p are predicted to bind CCND1, CCND2, CDK7, E2F1, E2F3, E2F3, and MYC mRNAs. Several studies have shown that these miRNAs play a role in the regulation of these genes (90–93). In the present study, the mRNA levels of CCND1, CCND2, CDK7, E2F1, E2F3, E2F3, and MYC were increased in male mice. As a downregulated miRNA usually associates with a target gene exhibiting increased expression, in male mice, it seems to be a concordance between the downregulation and upregulation of miRNAs and target genes, respectively, in response to infection and infection plus SP-A2 (1A^0^) protein rescue. In female mice, however, despite the miRNA downregulation, there was a decreased expression of these genes. These indicate that different mechanisms may be operative in the LAC miRNome in male and female mice, as also observed in previous studies (53, 55, 56).

The pro-inflammatory responses were mediated *via* STAT-3 and NF-κB. These pathways have been shown to be involved in inflammatory processes and lung disease (115–120). The expression of miR-17-5p, predicted to bind and regulate STAT-3, was significantly decreased, and this was associated with an increased expression of STAT-3 in male mice. Previously, we have shown that STAT-3 levels were significantly increased after 4 h of post-oxidative stress due to ozone exposure in male AMs from mice expressing the human SP-A2 (1A^0^) transgene (53) or after 4 h following infection in mice expressing the human SP-A (1A^0^) or both human SP-A transgenes (55). Whereas after 18 h following infection, the STAT-3 levels were also increased in female mice (55, 56), indicating a time-dependent and sex-specific regulation of STAT-3. Furthermore, in male mice, the level of EGR2 that contributes, *via* STAT-3, to the upregulation of pro-inflammatory cytokines was also upregulated compared to female mice. These data indicate that the NF-κB and STAT-3-mediated pathways are important in the pro-inflammatory gene expression in KO male mice. However, in female mice, these pathways may be compromised. Moreover, the role of the sex-dependent miRNA-target genes in the regulation of the inflammatory response to infection in the presence or absence of SP-A warrants further investigation.

### Lung Alveolar Cell Knockout Gene Expression in Response to Infection or Infection Plus SP-A Rescue

The cell cycle signaling node and the TP-53 node were found to be important in the gene expression study of both groups, infection and infection plus rescue. The former has been discussed above. The TP-53 node was unexpected, as this one is shown to associate with lung cancer (121). SP-A1 (6A^4^) and SP-A2 variants have been shown previously to associate with lung carcinoma (122–124). Although SP-A may regulate the tumor microenvironment *via* its ability to modulate cytokine expression and the polarization of macrophages in lung cancer (125), given the short time interval (6 h) in the present study from infection to data analysis, the changes in the expression of these various genes are likely due to bacterial infection. It is unlikely that this could be due to any true carcinogenic modulation, as this would require a considerably longer time than the 6-h time point used here. Several of the identified genes from the gene expression study had direct interaction with TP-53 in response to infection and infection plus SP-A2 (1A^0^) protein rescue. In response to infection, in male mice, the TACC2, BUB1B, ATP5MC3, and MYO1E, and in female mice, the CAP1, MRPL2, COX5A, KLHL21, PDIA6, TSPO, and USP14 had direct interaction with TP-53 (**Supplementary Figure 1**). In response to SP-A2 (1A^0^) protein rescue in male mice, the PGD, NAB1, RPL10, CDC42, and PDCD6IP had direct interaction with TP-53, whereas the NFAM1, PSMC1, and TP2B had indirect interaction with TP-53 (**Supplementary Figure 1**). In female mice, MDH2, NDUFS6, CSTB, LRRC17, TALDO1, CTSD, PFN1, S100A4, FAM120A, HUWE1, MCAM, GLUL, and UQCRQ had direct interaction with TP-53 (**Supplementary Figure 1**). These indicate that regardless of the role of TP-53 in infection, significant differences exist in the specific genes involved and the number of genes that interact with TP-53 as a function of sex, infection, and SP-A genotype that warrant further investigation.

## Comments and Summary

A number of studies with similar mouse models exposed to different insults have, previously, been carried out. In the grand scheme of things, similar observations were made in terms of pathways involved, albeit with some differences among the molecules involved. In the previously published mouse models where the miRNome and signaling pathways were studied, the mice were exposed to ozone and these were chronically/constitutively exposed to SP-A (53–56), whereas in the current study, the SP-A-KO mice were never exposed to SP-A until experimentation where they were acutely treated with SP-A at the time of infection. Interestingly, regardless of the insult/exposure (i.e., infection or ozone exposure), a number of similarities in the general response were observed. For example, after infection or ozone exposure, miRNAs were largely decreased in male and female mice, although differences in the levels of their target genes in terms of increases or decreases were observed between males and females. Even though LACs after infection in the present SP-A rescue study consist of ~70%–75% AMs and LACs after ozone exposure of humanized transgenic mice exposed chronically to human SP-A1 and SP-A2 constist of ~95% AMs (53, 55), following IPA, some of the signaling pathways were similar for the miRNA-mRNA targets regardless of exposure conditions.

Knowing the pathways and molecules involved in response to infection after an acute treatment of SP-A-KO mice with SP-A may have clinical importance if SP-A is used as a therapy. One potential example is the prematurely born infant who has low levels of SP-A, and infection has been identified as a major complication in these infants (126–128). Other conditions that may benefit from SP-A therapy include RSV and asthma, where SP-A or specific fragments/peptides of SP-A have already been used in preclinical studies as a potential therapy (57, 58). The present study provides insights that may be useful, as considerations for the therapeutic value of SP-A may expand in the future.

In related mouse models (regardless of the type of insult) where the miRNome and/or the gene expression profile of the AM or of the LAC was studied, two of the signaling pathways that were found to be significant in more than one study were rather unexpected. One of these was TP-53, which is shown to associate with lung cancer and is discussed in the previous section. Another one was the cell cycle signaling pathway. This pathway was ubiquitous, as this was not only significant in all comparisons made in the current study but in previously published studies of related mouse models (42, 55, 56). This observation, although it is surprising and difficult to explain because the AM (the predominant cell in LACs 6 h after infection or after 4 h of ozone exposure), and macrophages, in general, are not known to multiply. However, a rather recent literature challenges this notion. In a recent review, Röszer (129) discusses the self-renewal of macrophages at various tissue locations. For AMs, in particular, local proliferation was noted in mice and humans under certain conditions, and this capacity of self-renewing was also demonstrated *in vitro* (129). A number of mitogenic signals have been shown to play a role in macrophage proliferation. These include macrophage colony-stimulating factor (M-CSF) and granulocyte-macrophage (GM)-CSF (130) and IL-1α (131). The latter in certain conditions modulates the proliferation of a subset of AMs (131). Of interest, IL-1α levels in BAL were shown to be increased after infection (132), and during asthmatic inflammation, the AM pool at the early stages of the process depends on local proliferation (133). Furthermore, because the cell cycle pathway has been observed not only after infection (present study) where AMs are ~70%–75% of the LAC but also after 4 h of ozone exposure where AMs are ~95% of the LAC (53, 55), it is likely that this pathway occurs in AMs rather than other LACs that may constitute a fairly small portion of the LACs. Although it is currently unknown whether infection under the studied conditions can cause local AM proliferation, this possibility, however, cannot be excluded at this point, as the present data provide a general support that this may occur. This is an interesting possibility and warrants further investigation.

The novelty of the present study is to the best of our knowledge that this is the first such study where the SP-A-KO mice were rescued with/exposed to SP-A at the time of infection and the LAC miRNome was studied. The similarity of molecules/pathways observed in response to SP-A among various studies, whether acute or chronic exposure (as discussed above), points to an important role of SP-A in LACs under various conditions. However, studying, as done here, LACs as a whole instead of a given cell type is a limitation, and in future studies, investigation of individual types of cells and the use of other methods is needed. For example, studying alveolar cells after cell sorting will better determine which miRNAs/pathways are attributed to AMs and which ones are attributed to other cells in BAL under the studied conditions.

In summary, the present study showed that 1) sex differences exist in all analyses performed; 2) the cell cycle pathway is significant in all study groups, although miRNAs and molecules involved in the cell cycle may differ under the different studied conditions; 3) pro-inflammatory pathways play an important role, and these may be more pronounced in the absence of SP-A; and 4) the gene expression profile identified the TP-53 and the cell cycle nodes as significant pathways. These unexpected findings are of particular interest and warrant further study. These together provide the foundation for future mechanistic studies where the details of SP-A-mediated pathways after bacterial infection could be investigated in a pure LAC population after employing cell sorting purification techniques (134).

## Data Availability Statement

The datasets presented in this study can be found in online repositories. The names of the repository/repositories and accession number(s) can be found in the article/**Supplementary Material**.

## Ethics Statement

The protocols used were approved by the Pennsylvania State University College of Medicine Institutional Animal Care and Use Committee. The content on the care and use of laboratory animals were according to the guidelines of the National Institutes of Health.

## Author Contributions

NT and JF designed the study. NT performed the experiments, data analysis, and synthesis and contributed to the article writing. JF provided oversight in data analysis and integration and in the writing of the article. All of the authors read and approved the final article.

## Funding

This work was supported by the John Ardell Pursley Memorial Research Fund and the Center for Host Defense, Inflammation and Lung Disease (CHILD) Fund, Department of Pediatrics, Penn State University College of Medicine.

## Conflict of Interest

The authors declare that the research was conducted in the absence of any commercial or financial relationships that could be construed as a potential conflict of interest.

## Publisher’s Note

All claims expressed in this article are solely those of the authors and do not necessarily represent those of their affiliated organizations, or those of the publisher, the editors and the reviewers. Any product that may be evaluated in this article, or claim that may be made by its manufacturer, is not guaranteed or endorsed by the publisher.

## Supplementary Material

Supplementary File 1 LAC KO miRNAs of both sexes at 6h after infection and after infection plus SP-A2 (1A^0^) protein rescue.

Supplementary File 2 LAC KO gene expression of both sexes at 6h after infection and after infection plus SP-A2 (1A^0^) protein rescue.

Supplementary Figure 1 Biological networks for the cell cycle and TP-53 signaling pathways of genes with ≥ 2-fold expression levels in both sexes are shown for the KO at 6 h after infection with or without SP-A2 (1A0) protein rescue. Direct and indirect gene interactions are marked with solid and dashed lines, respectively. Networks on the left and right sides of the figure show pathways for male and female mice, respectively. Each gene or group of genes is represented as a node. The different shapes depict different functional classes as follows: Square and concentric (double) circles denote cytokines and complex/groups, respectively. Diamonds denote peptidases and enzymes; Ovals denote transmembrane receptors and transcription regulators; Triangle kinases and phosphatases; Rectangles ion channels, Gprotein coupled receptors, and ligand-dependent nuclear receptors; and Trapezoids microRNAs and transporters.

## Glossary

**Table:**

ACAA1b	acetyl-CoA acyltransferase 1
AM	alveolar macrophage
ANOVA	analysis of variance
ARPC1B	actin-related protein 2/3 complex subunit 1B
ATP6V0D2	ATPase H+ transporting V0 subunit D2
ATP5MC3	ATP synthase membrane subunit C locus 3
BAL	bronchoalveolar lavage
BCL2	B-cell lymphoma 2
BUB1B	mitotic checkpoint serine/threonine kinase B
CAP1	cyclase-associated actin cytoskeleton regulatory protein 1
CASP9	caspase 9
CCND1	cyclin D1
CCND2	cyclin D2
CDC42	cell division cycle 42
CDK7	cyclin-dependent kinase 7
CDKN2A	cyclin-dependent kinase inhibitor 2
COTL1	coactosin-like F-actin binding protein 1
COX5A	cytochrome C oxidase subunit 5A
CSTB	cystatin B
CTSD	cathepsin D
DBI	diazepam-binding inhibitor acyl-CoA binding protein
E2F1	E2F transcription factor 1
E2F2	E2F transcription factor 2
E2F3	E2F transcription factor 3
EGR2	early growth response 2
FAM120A	family with sequence similarity 120A
FOXO1	Forkhead box O1
FOXO3	Forkhead box O3
GAPDH	glyceraldehyde 3-phosphate dehydrogenase
hTG	humanized transgenic
HUWE1	HECT UBA and WWE domain-containing E3 ubiquitin protein ligase 1
GLUL	glutamate-ammonia ligase
IL-6	interleukin 6
IPA	Ingenuity Pathway Analysis
IVNS1ABP	influenza virus NS1A-binding protein
KLHL21	Kelch-like family member 21
KO	knockout
LAC	lung alveolar cell
LITAF	lipopolysaccharide-induced TNF
LRRC17	leucine-rich repeat containing 17
MBNL1	muscleblind-like splicing regulator 1
MCAM	melanoma cell adhesion molecule
MDH2	malate dehydrogenase 2
miRNAs	microRNAs
MRPL2	mitochondrial ribosomal protein L2
MYC	MYC proto-oncogene
MYO1E	myosin IE
NAB1	NGFI-A-binding protein 1
NDUFA7	NADH:ubiquinone oxidoreductase subunit A7
NDUFS6	NADH:ubiquinone oxidoreductase subunit S6
NFAM1	NFAT-activating protein with ITAM motif 1
PANK3	pantothenate kinase 3
PDIA6	protein disulfide isomerase family A member 6
PDCD6IP	programmed cell death 6-interacting protein
PFN1	profilin 1
PGD	phosphogluconate dehydrogenase
PIM1	proto-oncogene serine/threonine kinase
PPT1	palmitoyl-protein thioesterase 1
PPARA	peroxisome proliferator-activated receptor alpha
PPARG	peroxisome proliferator-activated receptor gamma
PRKAR1A	protein kinase CAMP-dependent Type I regulatory subunit alpha
PSMC1	proteasome 26S subunit ATPase 1
RNF187	ring finger protein 187
RPL4	ribosomal protein L4
RPL6	ribosomal protein L6
RPL10	ribosomal protein L10
RPL31	ribosomal protein L31
RPL35A	ribosomal protein L35a
RPL36a	ribosomal protein L36a
RPL37	ribosomal protein L37
RPS3	ribosomal protein S3
RPS9	ribosomal protein S9
RPS21	ribosomal protein S21
RPS25	ribosomal protein S25
SCARB2	scavenger receptor Class B Member 2
S*FTPA*1	gene-encoding SP-A1
S*FTPA2*	gene-encoding SP-A2
SMAD2	SMAD family member 2
SNAP23	synaptosome-associated protein 23
SP-A	surfactant protein A
SRGN	serglycin
STAT-3	signal transducer and activator of transcription 3
S100A4	S100 calcium-binding protein A4
TACC2	transforming acidic coiled-coil containing protein 2
TALDO1	transaldolase 1
TBCA	tubulin-folding cofactor A
TLR2	Toll-like receptor 2
TNF	tumor necrosis factor
TNFSF12	TNF super family member 12
TP-53	tumor protein 53
TSPO	translocator protein
UQCRQ	ubiquinol-cytochrome C reductase complex III subunit VII
UQCR11	ubiquinol-cytochrome C reductase complex III subunit XI
USP14	ubiquitin-specific peptidase 14
