# Supplementary material for: The Lung Alveolar Cell (LAC) miRNome and Gene Expression Profile of the SP-A-KO Mice After Infection With and Without Rescue With Human Surfactant Protein-A2 (1A0)
Source: Front Immunol. 2022 Jul 1;13:854434. doi: 10.3389/fimmu.2022.854434 (PMC9283764; doi:10.3389/fimmu.2022.854434)

Supplementary Figure 1

Infection

Cell cycle signaling node

Males

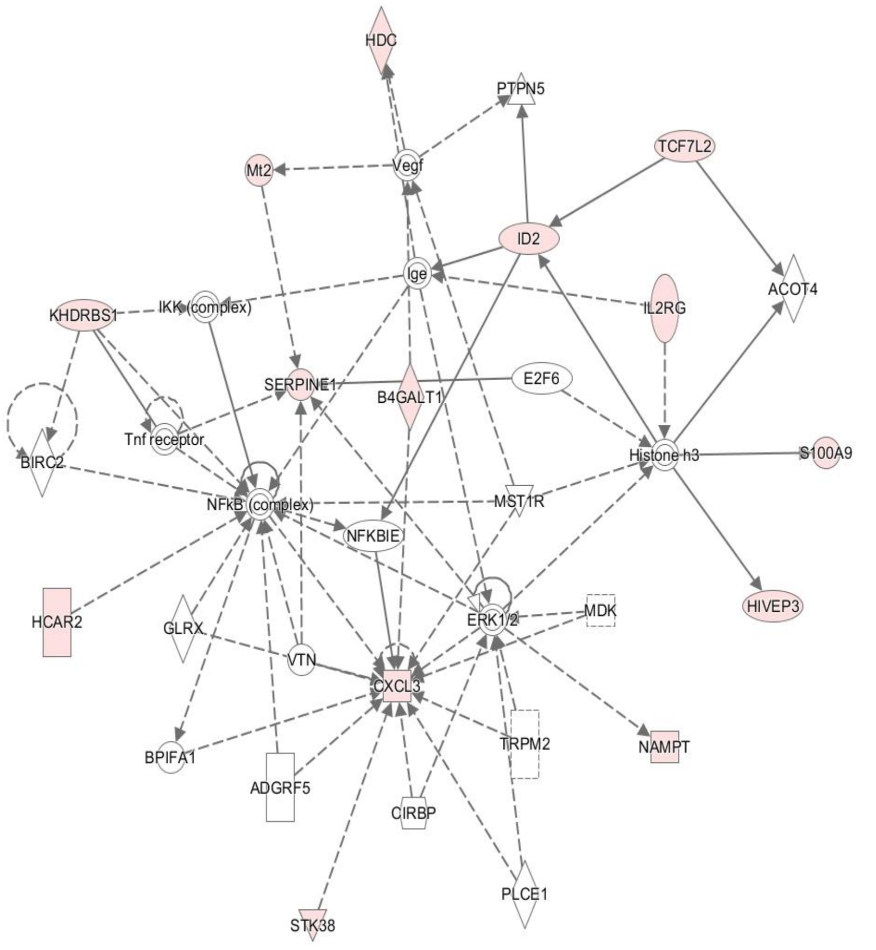

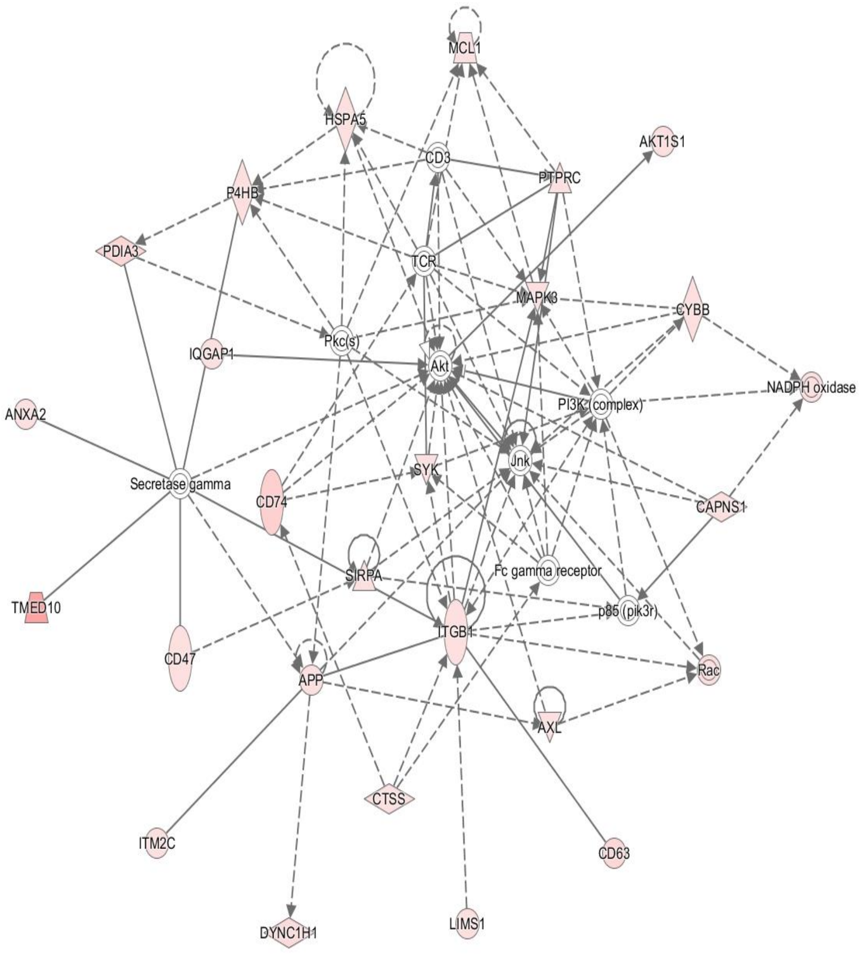

Females

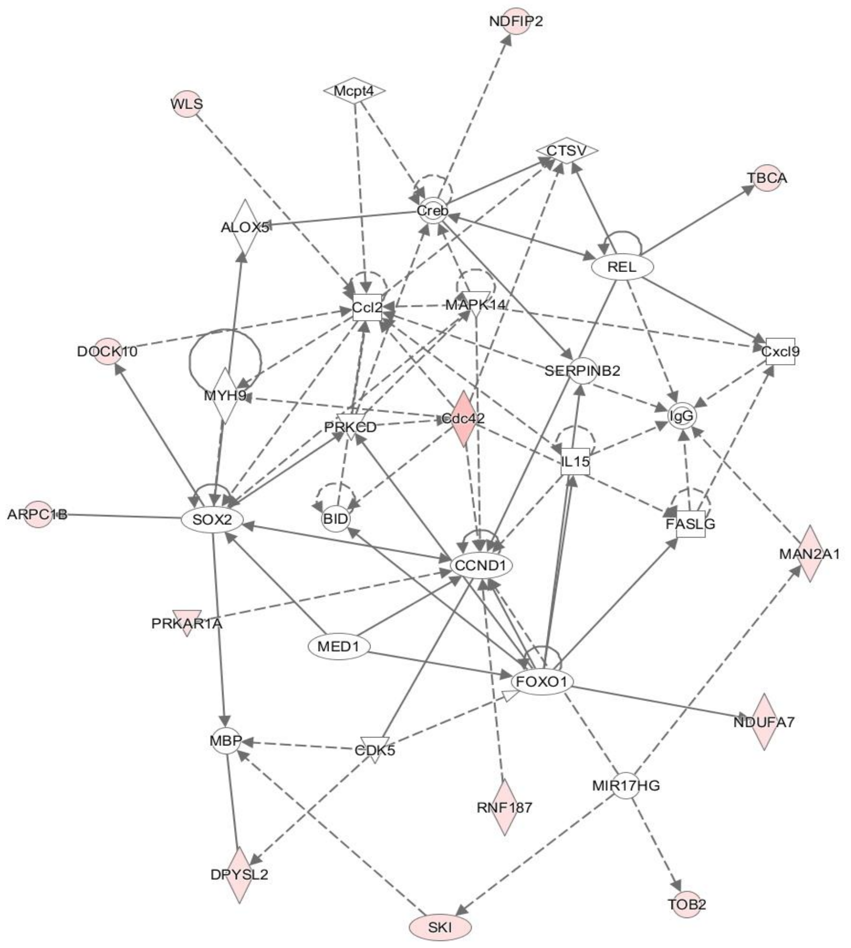

Infection

TP-53 node

Males

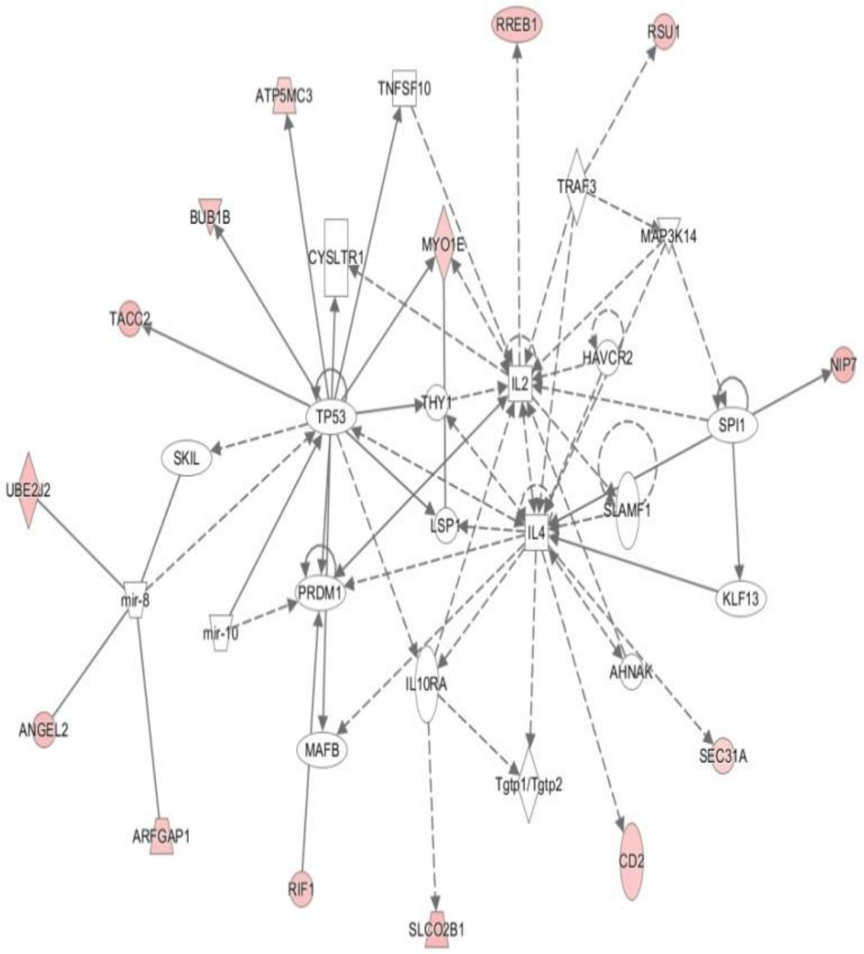

Females

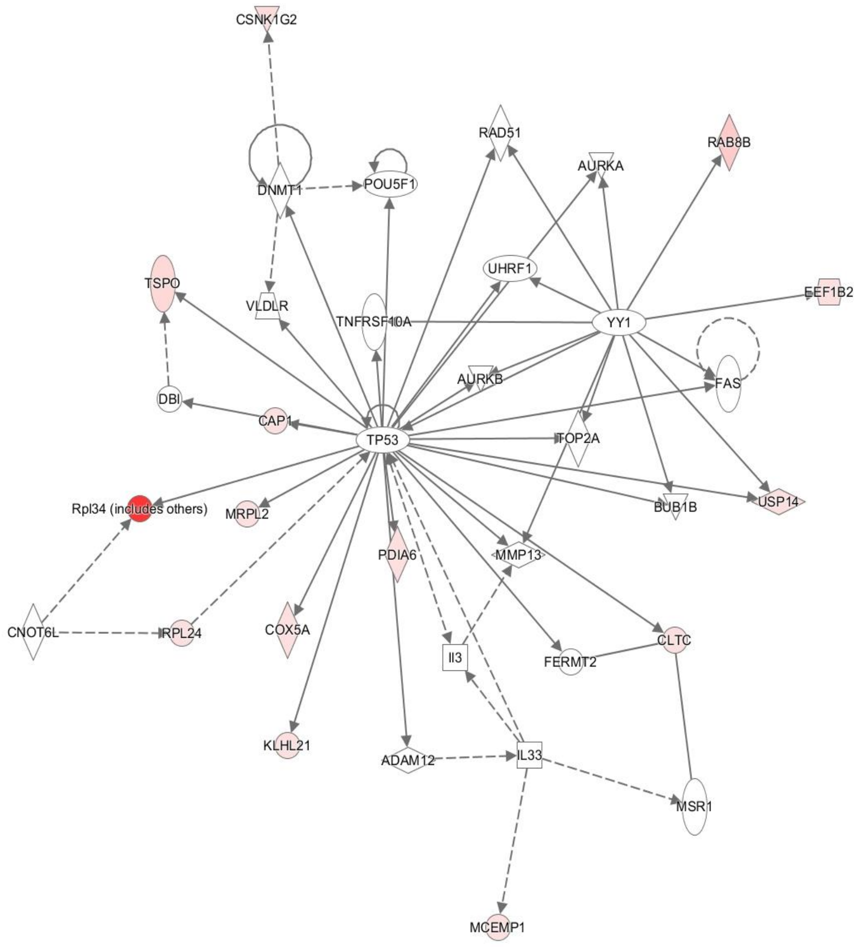

Infection + SP-A2(1A^0) protein

Cell cycle signaling node

Males

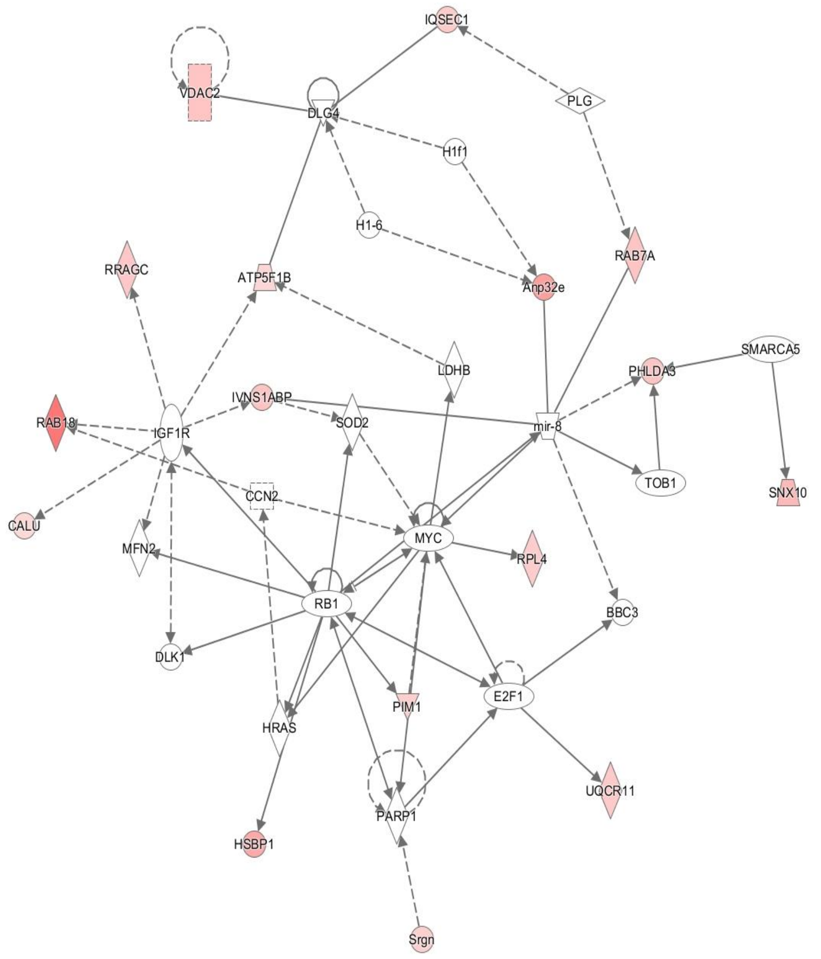

Females

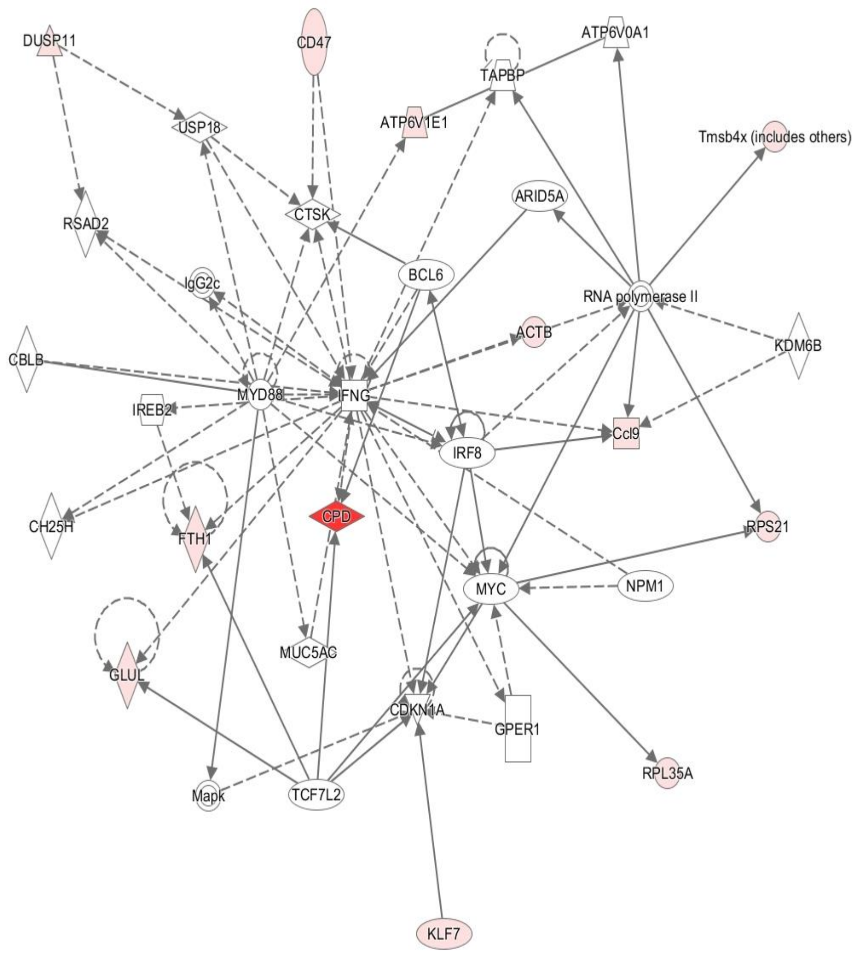

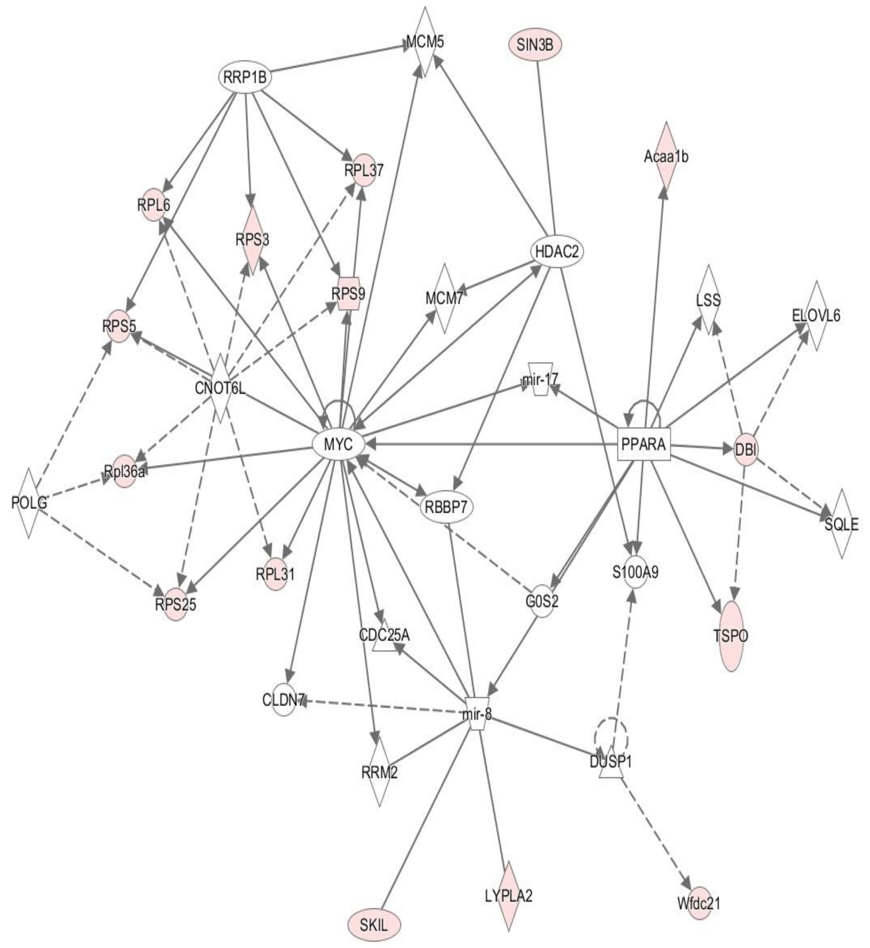

Infection + SP-A2(1A^0) protein

TP-53 node

Males

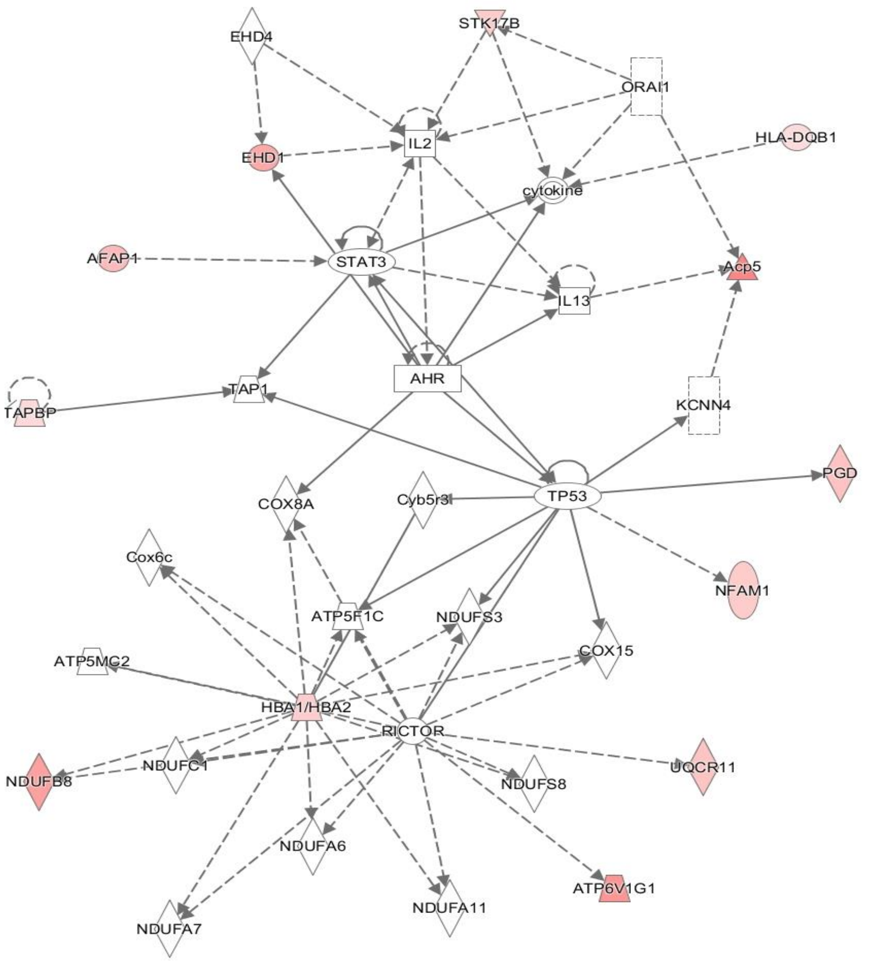

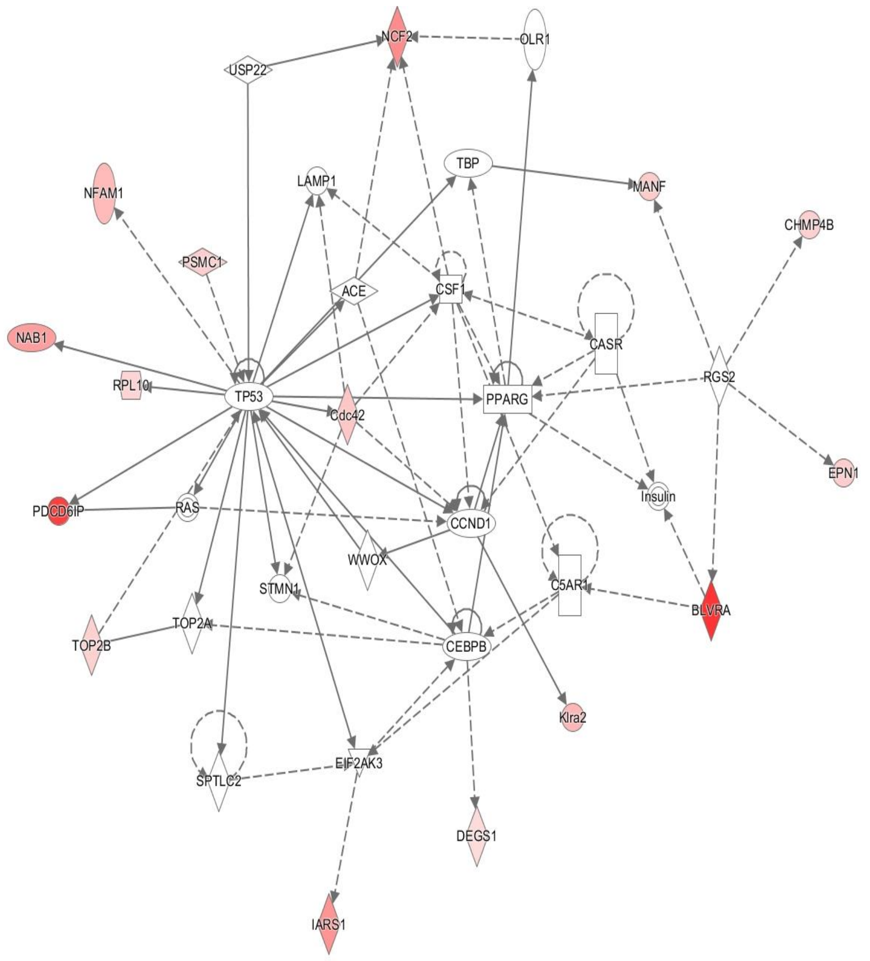

Females

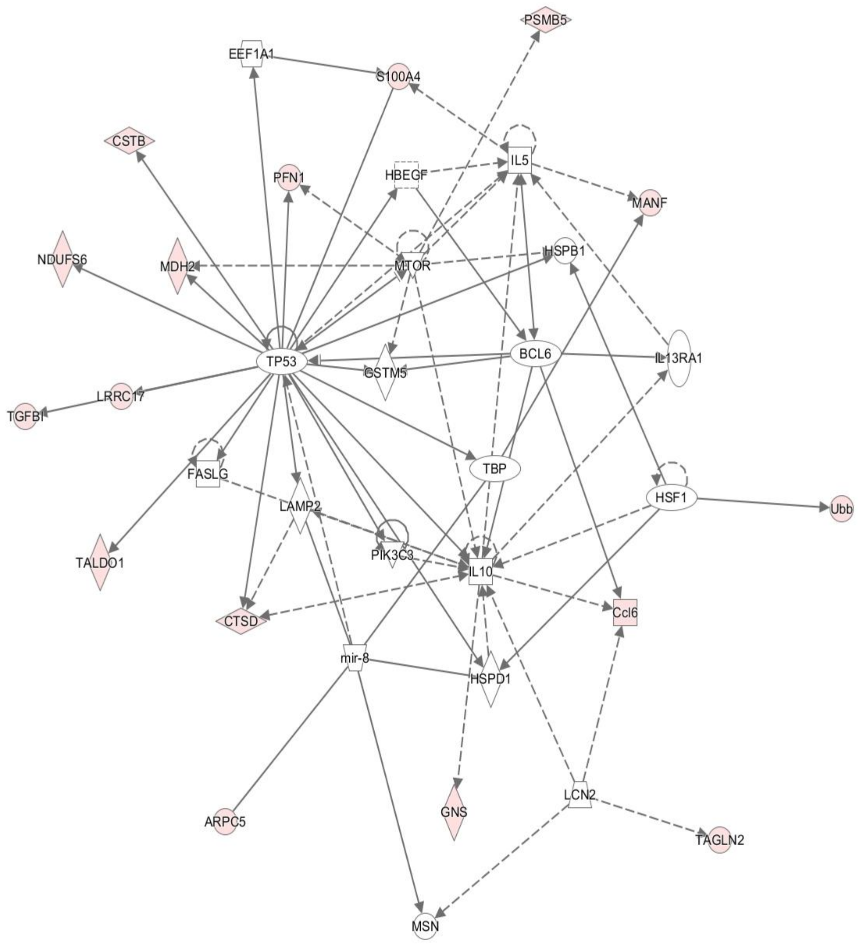

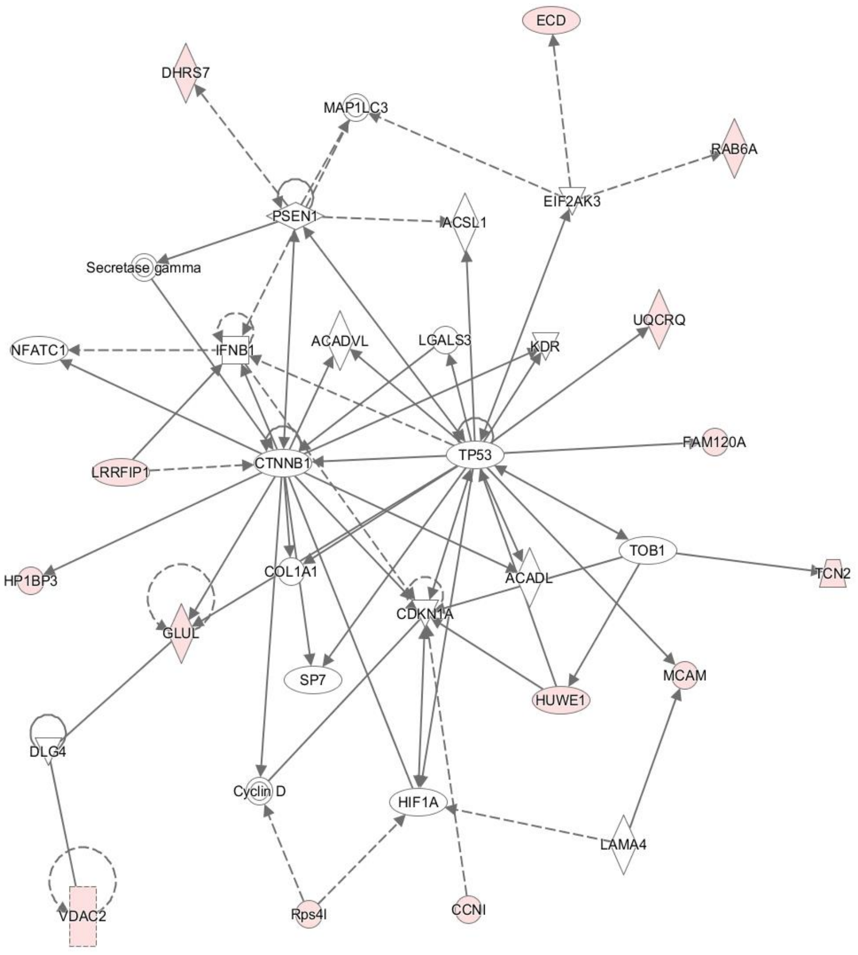

Supplement: Supplementary Figure 1 — Biological networks for the cell cycle and TP-53 signaling pathways of genes with ≥ 2-fold expression levels in both sexes are shown for the KO at 6 h after infection with or without SP-A2 (1A0) protein rescue. Direct and indirect gene interactions are marked with solid and dashed lines, respectively. Networks on the left and right sides of the figure show pathways for male and female mice, respectively. Each gene or group of genes is represented as a node. The different shapes depict different functional classes as follows: Square and concentric (double) circles denote cytokines and complex/groups, respectively. Diamonds denote peptidases and enzymes; Ovals denote transmembrane receptors and transcription regulators; Triangle kinases and phosphatases; Rectangles ion channels, Gprotein coupled receptors, and ligand-dependent nuclear receptors; and Trapezoids microRNAs and transporters. [file DataSheet_3.pdf]
